# Disparities in the incidence, mortality and disability-adjusted life years of 33 early-onset cancer groups globally, 2012–2021: a systematic analysis

**DOI:** 10.1186/s40164-025-00634-7

**Published:** 2025-03-17

**Authors:** Wenxin Yan, Min Liu, Wenzhan Jing, Liangyu Kang, Ning Zhang, Haoran Sun, Jinyu He, Zhongdan Chen, Jue Liu, Wannian Liang, Jiahong Dong

**Affiliations:** 1 Vanke School of Public Health, Tsinghua University, Beijing, China; 2 School of Public Health, Peking University, Beijing, China; 3 Department of Surgery, Asian Liver Center, Stanford University School of Medicine, Palo Alto, CA USA; 4 World Health Organization Representative Office for China, Beijing, China; 5 Institute for Healthy China, Tsinghua University, Beijing, China; 6 School of Clinical Medicine, Key Laboratory of Digital Intelligence, Hepatology (Ministry of Education), Tsinghua University, Beijing, China

**Keywords:** Early-onset cancer, GBD, Incidence, DALY

## Abstract

**Background:**

The global cancer burden is rising, with early-onset cancers becoming more prevalent. We aimed to investigate the burden, trend and population disparity in 33 early-onset cancers from 2012 to 2021.

**Methods:**

Annual incidence, death, and disability-adjusted life years (DALY) numbers and rates for early-onset (15–49 years) cancer groups were calculated from Global Burden of Diseases (GBD) 2021 dataset, covering 2012–2021 across global, five SDI groupings, and 204 countries and territories. Estimated annual percentage change (EAPC) in the incidence, mortality and DALY rates was calculated to quantify temporal trends, while spearman correlation analysis was used to examine the correlation between rates, EAPC and SDI.

**Results:**

In 2021, there were 2.65 million new early-onset cancer cases excluding non-melanoma skin cancer (NMSC), resulting in 0.99 million deaths and 50.7 million DALYs. Breast, tracheal, bronchus and lung (TBL), cervical, colon and stomach cancers were the leading causes of DALYs. The DALY rate for early-onset cancer excluding NMSC changed from 65.7 million in 2012 to 67.0 million in 2021, with an estimated annual percentage change (EAPC) of -0.49%. While the DALY rate plateaued for females, it decreased by -0.95% for males. Ten of 33 cancer groups exhibited an EAPC > 0. The high SDI quintile had 1,100 DALYs per 100,000 caused by early-onset cancers excluding NMSC, with the highest declining trend in DALY and mortality rates, while the high-middle SDI quintile had the highest early-onset mortality rates. Rising trends in cancer incidence and mortality were especially notable among females in the middle, low-middle, and low SDI quintiles.

**Conclusion:**

The global burden of early-onset cancer differs significantly by SDI quintile and gender. The increasing burden across multiple cancer groups poses a significant public health challenge. The rising burden of multiple cancer types is alarming, highlighting the need for increased policy support and targeted medical assistance to address the disparities in their impact.

**Supplementary Information:**

The online version contains supplementary material available at 10.1186/s40164-025-00634-7.

## Introduction

Cancer poses a significant global public health challenge and is a major contributor to the global disease burden, with projections indicating that this burden will continue to rise for at least the next two decades [1, 2]. This situation imposes a serious burden on individuals, families, and society as a whole [3]. The United Nations (UN) Sustainable Development Goals (SDGs) acknowledge the necessity of addressing cancer burden under target 3.4, which states, “By 2030, reduce by one-third premature mortality from noncommunicable diseases (NCDs) through prevention and treatment and promote mental health and well-being.” [4] Most countries must enhance their efforts to alleviate the cancer burden in order to achieve this target [5]. In recent years, progress has been made in cancer prevention and treatment, leading to reductions in mortality rates for certain cancers. However, this increase in global cancer burden may be due to factors such as population aging, growth, and an increasing prevalence of risk factors such as smoking, obesity, and physical inactivity [6, 7]. Concurrently, it is concerning that recent studies have emphasized the rising alarm over early-onset cancers, defined as those diagnosed in individuals under the age of 50. In 2019, the global incidence of early-onset cancers exceeded 3.26 million cases, marking a 79.1% increase since 1990 [8]. 

This trend is particularly troubling, as early-onset cancers significantly affect individuals, families, and society due to the loss of productive years of life. Notably, the incidence of breast, lung, and colorectal cancers has risen globally among individuals under 50, particularly affecting women [9–11]. While mortality rates for these cancers have declined, the total number of deaths has nonetheless increased [12]. Research shows that younger women tend to experience more aggressive forms of breast cancer and worse outcomes compared to their older counterparts. Additionally, research indicates that advancements in conditional survival for colorectal cancer (CRC) are less significant in younger individuals compared to older adults [13]. The rising incidence of cancer among younger populations has heightened the demand for early screening measures. Advocates have actively promoted initiatives targeting individuals under the age of 50. In 2018, the American Cancer Society recommended that individuals start colorectal cancer screening at age 45, changing the previous guideline of age 50 [12]. To effectively guide practice, it is essential to examine the burden and changing trends of various cancer types in recent years. Consequently, gaining insights into the latest trends in early-onset cancer is vital, as is identifying which specific cancers need attention and evaluating the degree of inequality in cancer burden.

Although numerous previous studies have focused on the changes in early-onset cancers over the past 30 years [8, 14] and the burden of cancer across the general population [15], a systematic analysis of the latest trends in early-onset cancers for all 33 types, including cancer spectrum analysis and population differences, still needs to be updated. This analysis can reveal variations in early-onset cancer incidence across different regions, genders, and socioeconomic backgrounds, aiding researchers in identifying potential environmental, genetic, and lifestyle factors, which can inform future public health policies.

## Methods

### Study design and data sources

Early-onset cancers were often defined as those that occur in adults under the age of 50 [12]. In this article, we aggregated the cancer burden in individuals aged 15–49 and defined this group as the early-onset population. We collected yearly numbers and rates on disability-adjusted life years (DALYs), incidence, and deaths of total and specific groups of cancers, within the age range of 15–49 years from 2012 to 2021 at country level from the Global Burden of Disease Results (https://vizhub.healthdata.org/gbd-results/), a widely used database coordinated by the Institute for Health Metrics and Evaluation (IHME) [16]. The GBD 2021, with the support of over 11,500 collaborators from 164 countries, employs a systematic methodology for the assessment of global health status and disease burden, based on extensive data provision, review, and analysis. The data sources can be found through the GBD 2021 Data Input Sources Tool (https://ghdx.healthdata.org/gbd-2021/sources) from IHME.

Disease and injuries in GBD 2021 were structured in a comprehensive, hierarchical system, with neoplasms classified as one of 22 level 2 groups [17]. Cancers were classified into 34 level 3 cancer groups (e.g., Liver cancer). The benign and in situ neoplasms are counted as the overall neoplasm burden, but not in total cancers [15]. The international classification of disease codes of these 33 cancers in GBD 2021 were defined in the supplementary appendix (Appendix 1). And similarly, because nonmelanoma skin cancer (NMSC) has relatively high incidence and low mortality compared with other cancers, we estimate with and without NMSC.

Results are presented by socio-demographic index (SDI), a composite indicator estimated to represent a comprehensive development status that exhibits a robust correlation with health outcomes [18–20]. It is derived from the geometric mean of 0–1 indices of the fertility rates among females under the age of 25, mean years of education for individuals aged 15 and above, and lag-distributed income per capita. While SDI values may change over time, for consistency of comparison, countries were grouped into quintiles according to their SDI values in 2021. These quintiles were termed high, high-middle, middle, low-middle, and low.

### Statistical analysis

We calculated the estimated annual percentage change (EAPC) in early-onset rate of mortality, incidence and DALY to evaluate the average changing trends over a specified time interval. We fitted the regression line: *γ* = *α* + *β*x + *ε*, where *β* represents the annual change in ln(early-onset rate) and calculated EAPC as 100×(e^*β*^–1), along with corresponding 95% confidence intervals [21]. If annual percentage change estimates and 95% confidence intervals were both > 0 (or both < 0), we considered the corresponding rate to be in an upward (or downward) trend.

We employed local regression smoothing models (loess) using “geom_smooth” function of package “ggplot2” to fit the correlation between the burdens and their EAPCs of early-onset cancers and SDI across 7 GBD super regions and 204 countries and territories (the super region of countries and territories were recorded in Table S1). We regarded *p* < 0.05 as statistically significant. All statistical analysis and graphical representations were conducted using R software (version 4.3.2) and GraphPad Prism (version 10.2.0).

### Ethics statement

Being involved in the Global Burden of Disease 2021 rather than directly speaking to patients inspired this research. Although no patient was directly involved in this study, members of the public read our manuscript, and all agreed on the specific findings of this study.

### Role of the funding source

The funders of the study had no role in study design, data collection, data analysis, data interpretation, or writing of the report. All authors had full access to all the data in the study and accepted responsibility to submit for publication.

## Results

### Global burden of 33 early-onset cancer groups in 2021

Across 204 countries and territories, there were 23.6 million (95% UI, 22.2–24.8 million) incident cancer cases, 9.83 million (95% UI, 9.07–10.5 million) deaths in 2021 among the general population (Table S2). Excluding NMSC, there were estimated 17.2 million (95% UI, 16.1–18.3 million) incident cancer cases, 9.78 million (95% UI, 9.02–10.5 million) deaths (Table S2). Total cancers and cancers excluding NMSC were estimated to cause 252 million (95% UI, 236–269 million) and 251 million (95% UI, 235–268 million) DALYs respectively.

In population aged 15–49 years, namely early-onset population, there were 3.16 million (95% UI, 2.98–3.34 million) incident early-onset cancer cases and 0.99 million (95% UI, 0.93–1.05 million) deaths in 2021, and early-onset cancers were estimated to cause 50.9 million (95% UI, 47.7–54.0 million) DALYs. Excluding NMSC, there were estimated 2.64 (2.50–2.81) million incident early-onset cancer cases and 0.99 (0.92–1.05) million deaths in 2021, and 50.7 (47.5–53.8) million DALYs caused by early-onset cancers (Table S2).

The 5 leading causes of early-onset cancer-related DALYs rate were breast cancer (170.8, 159.1-183.3 per 100,000); tracheal, bronchus, and lung cancer (119.6, 106.5-132.5); cervical cancer (106.0, 95.7-117.2); colon and rectum cancer (101.4, 92.9-110.2); and stomach cancer (97.7, 85.0-112.2). Among males, the leading causes of early-onset cancer-related DALYs globally were TBL, stomach, colon and rectum, liver cancer, and leukemia. Among females, the leading causes of early-onset cancer-related DALYs globally were breast, cervical, TBL cancer, leukemia, and colon and rectum cancer (Table 1).

**Table 1:** Global early-onset DALY, incidence and mortality rate in 2021 for total cancers excluding NMSC and 33 cancer groups

Cancer type	DALY rate per 100 000 (95% UI)			Incidence rate per 100 000 (95% UI)			Mortality rate per 100 000 (95% UI)		
	Total	Male	Female	Total	Male	Female	Total	Male	Female
Total Cancers excluding Non-melanoma skin cancer	1284 (1203–1361)	1220 (1116–1335)	1349 (1246–1454)	67.0 (63.4–71.1)	51.2 (47.1–55.8)	83.3 (77.6–89.7)	25.0 (23.4–26.5)	24.0 (21.9–26.3)	26.0 (24.1–28.0)
Breast cancer	171 (159–183)	4.18 (2.76–5.60)	342 (318–367)	14.4 (13.4–15.5)	0.323 (0.189–0.412)	28.8 (26.8–30.9)	3.32 (3.09–3.57)	0.081 (0.054–0.108)	6.64 (6.17–7.13)
Tracheal, bronchus, and lung cancer	120 (106–132)	153 (132–176)	85.8 (76.1–97.4)	3.13 (2.78–3.47)	3.88 (3.34–4.48)	2.35 (2.08–2.69)	2.51 (2.23–2.78)	3.21 (2.77–3.71)	1.79 (1.59–2.03)
Cervical cancer	106 (95.7–117)	NA	215 (194–238)	7.79 (7.11–8.50)	NA	15.8 (14.4–17.2)	2.07 (1.87–2.29)	NA	4.19 (3.79–4.64)
Colon and rectum cancer	101 (92.9–110)	119 (105–134)	82.9 (75.7–90.7)	5.37 (4.91–5.86)	6.33 (5.5–7.23)	4.37 (4.00-4.79)	2.01 (1.84–2.19)	2.37 (2.07–2.67)	1.65 (1.51–1.8)
Stomach cancer	97.7 (85–112)	121 (99.5–147)	74.3 (66.8–82.4)	3.17 (2.72–3.67)	4.09 (3.34–5.06)	2.22 (1.99–2.5)	2 (1.74–2.3)	2.49 (2.06–3.05)	1.49 (1.34–1.65)
Leukemia	96.3 (78.3–108)	108 (78.9–130)	84.7 (66-94.3)	2.4 (1.96–2.68)	2.73 (1.98–3.27)	2.06 (1.61–2.32)	1.69 (1.38–1.89)	1.89 (1.39–2.29)	1.49 (1.16–1.66)
Brain and central nervous system cancer	74.5 (62.8–88.1)	86 (64.5–110)	62.8 (55.3–70.7)	2.47 (2.11–2.87)	2.69 (2.04–3.38)	2.24 (1.99–2.57)	1.39 (1.17–1.64)	1.61 (1.21–2.06)	1.16 (1.02–1.30)
Liver cancer	73.2 (64–85)	112 (95.1–136)	33.2 (29.9–37.1)	1.9 (1.65–2.22)	2.95 (2.5–3.6)	0.816 (0.730–0.915)	1.49 (1.30–1.74)	2.3 (1.95–2.79)	0.662 (0.594–0.738)
Non-Hodgkin lymphoma	54.8 (49.3–61.7)	66.5 (59.7–76.9)	42.7 (37.4–48.2)	2.95 (2.71–3.19)	3.67 (3.3–4.07)	2.21 (2.00-2.44)	0.991 (0.893–1.11)	1.21 (1.09–1.41)	0.765 (0.672–0.857)
Other malignant neoplasms	41.8 (36.5–46.1)	43.5 (35.5–50.2)	40.0 (34.7–46.9)	1.89 (1.67–2.06)	1.85 (1.55–2.10)	1.94 (1.67–2.19)	0.78 (0.68–0.85)	0.81 (0.67–0.94)	0.74 (0.64–0.87)
Esophageal cancer	39.3 (35.2–44.1)	59.3 (51.7–67.6)	18.8 (15.9–22.6)	1.08 (0.97–1.21)	1.64 (1.42–1.89)	0.509 (0.429–0.605)	0.834 (0.747–0.936)	1.27 (1.1–1.45)	0.391 (0.331–0.468)
Lip and oral cavity cancer	38.5 (33-42.6)	50.7 (41.9–56.9)	26.0 (22.3–30.2)	1.97 (1.73–2.14)	2.47 (2.11–2.73)	1.46 (1.29–1.66)	0.767 (0.662–0.846)	1.02 (0.849–1.15)	0.504 (0.437–0.584)
Ovarian cancer	32.8 (28.9–36.2)	NA	66.4 (58.5–73.4)	2.17 (1.90–2.41)	NA	4.4 (3.86–4.88)	0.64 (0.564–0.706)	NA	1.30 (1.14–1.43)
Pancreatic cancer	32.5 (29.5–35.7)	43.4 (38.7–48.7)	21.4 (19.5–23.2)	0.799 (0.726–0.874)	1.06 (0.947–1.19)	0.532 (0.487–0.575)	0.684 (0.62–0.75)	0.913 (0.813–1.03)	0.449 (0.410–0.487)
Malignant neoplasm of bone and articular cartilage	29.6 (25.1–34.6)	36.7 (28.0-44.2)	22.4 (18.9–27.4)	0.795 (0.659–0.902)	0.987 (0.765–1.17)	0.598 (0.511–0.704)	0.488 (0.410–0.568)	0.603 (0.460–0.720)	0.369 (0.315–0.444)
Nasopharynx cancer	23.4 (20.6–26.1)	32.6 (27.9–37.5)	13.9 (12.3–16.2)	1.16 (1.01–1.34)	1.65 (1.39–1.95)	0.656 (0.562–0.788)	0.455 (0.401–0.508)	0.639 (0.547–0.74)	0.266 (0.235–0.307)
Hodgkin lymphoma	17.3 (12.2–22.9)	20.6 (13.4–29.8)	13.9 (9.02–18.2)	0.901 (0.733–1.08)	1.03 (0.789–1.32)	0.766 (0.592–0.909)	0.291 (0.206–0.388)	0.351 (0.229–0.509)	0.229 (0.148–0.299)
Soft tissue and other extraosseous sarcomas	14.9 (12.7–19.1)	15.8 (12.5–22.9)	14.0 (11.9–18.3)	0.637 (0.545–0.794)	0.687 (0.559–0.936)	0.586 (0.508–0.751)	0.268 (0.228–0.342)	0.286 (0.226–0.412)	0.25 (0.211–0.326)
Other pharynx cancer	14.8 (13.2–16.3)	22.6 (20-25.7)	6.71 (5.43–8.49)	0.606 (0.555–0.658)	0.926 (0.846–1.02)	0.277 (0.234–0.344)	0.311 (0.277–0.344)	0.483 (0.426–0.547)	0.13 (0.11–0.17)
Kidney cancer	14.2 (13.2–15.1)	19.8 (18.3–21.6)	8.32 (7.66–9.05)	1.33 (1.26–1.41)	1.74 (1.61–1.89)	0.917 (0.851–0.996)	0.278 (0.260–0.296)	0.395 (0.365–0.430)	0.158 (0.146–0.171)
Malignant skin melanoma	12.3 (9.98–14.1)	12.9 (10.5–15.2)	11.6 (8.97–14.4)	1.69 (1.53–1.78)	1.46 (1.30–1.59)	1.93 (1.72–2.10)	0.226 (0.183–0.259)	0.243 (0.196–0.288)	0.209 (0.160–0.260)
Larynx cancer	11.8 (10.8–13.2)	18.9 (17.1–21.2)	4.57 (3.66–5.66)	0.467 (0.428–0.512)	0.754 (0.691–0.832)	0.171 (0.142–0.208)	0.248 (0.225–0.275)	0.400 (0.362–0.449)	0.091 (0.073–0.112)
Testicular cancer	11.7 (11.0-12.5)	23.1 (21.7–24.7)	NA	1.93 (1.86–2.02)	3.82 (3.67-4.00)	NA	0.187 (0.176–0.199)	0.370 (0.348–0.392)	NA
Gallbladder and biliary tract cancer	10.7 (8.42–12.4)	9.79 (6.39–11.6)	11.5 (8.79–14.2)	0.345 (0.27–0.4)	0.346 (0.231–0.414)	0.343 (0.267–0.418)	0.222 (0.176–0.259)	0.204 (0.134–0.242)	0.241 (0.184–0.295)
Uterine cancer	9.46 (7.83–10.7)	NA	19.2 (15.9–21.7)	1.49 (1.29–1.66)	NA	3.02 (2.60–3.36)	0.181 (0.152–0.204)	NA	0.368 (0.307–0.413)
Thyroid cancer	8.51 (7.11–10.1)	6.47 (5.31–7.43)	10.6 (8.5–13.5)	2.44 (2.13–2.79)	1.44 (1.23–1.61)	3.47 (2.93–4.20)	0.139 (0.119–0.162)	0.112 (0.092–0.128)	0.167 (0.135–0.210)
Bladder cancer	8.21 (7.42–9.11)	11.5 (10.1–13.4)	4.80 (4.31–5.35)	0.786 (0.718–0.869)	1.17 (1.03–1.33)	0.396 (0.361–0.44)	0.160 (0.145–0.177)	0.226 (0.198–0.26)	0.093 (0.083–0.103)
Multiple myeloma	7.45 (5.97–8.78)	8.7 (6.54–10.2)	6.16 (4.52–7.49)	0.247 (0.202–0.285)	0.286 (0.219–0.331)	0.208 (0.157–0.252)	0.153 (0.124–0.181)	0.178 (0.135–0.211)	0.126 (0.094–0.154)
Non-melanoma skin cancer	4.74 (3.97–5.28)	5.42 (3.99–6.33)	4.06 (3.55–4.57)	12.9 (10.6–15.2)	11.4 (9.22–13.5)	14.4 (12.0–17.0)	0.093 (0.077–0.104)	0.106 (0.078–0.124)	0.079 (0.069–0.090)
Prostate cancer	3.69 (2.94–4.17)	7.29 (5.8–8.24)	NA	0.452 (0.396–0.493)	0.893 (0.781–0.974)	NA	0.072 (0.057–0.082)	0.143 (0.113–0.162)	NA
Mesothelioma	3.2 (2.85–3.55)	3.94 (3.44–4.52)	2.43 (2.16–2.80)	0.078 (0.070–0.086)	0.097 (0.085–0.111)	0.059 (0.053–0.067)	0.064 (0.057–0.071)	0.080 (0.070–0.092)	0.048 (0.042–0.055)
Eye cancer	1.79 (1.27–2.37)	1.69 (0.953-2.8)	1.9 (1.34–2.81)	0.182 (0.129–0.248)	0.172 (0.103–0.267)	0.192 (0.13–0.272)	0.033 (0.023–0.044)	0.031 (0.018–0.051)	0.034 (0.024–0.050)
Neuroblastoma and other peripheral nervous cell tumors	1.49 (1.31–1.64)	1.62 (1.46–1.77)	1.37 (1.09–1.68)	0.051 (0.042–0.060)	0.056 (0.046–0.066)	0.046 (0.035–0.058)	0.026 (0.023–0.029)	0.029 (0.026–0.032)	0.024 (0.019–0.029)

Note: DALY = disability-adjusted life year

Among 22 groups of diseases and injuries in level 2 of the GBD cause hierarchy, total cancer was sixth-highest cause of DALYs behind respiratory infections and tuberculosis, mental disorders, musculoskeletal disorders, cardiovascular diseases, and self-harm and interpersonal violence (Figure S1).

### Global trends in cancer burden from 2012 to 2021

**Fig. 1:**
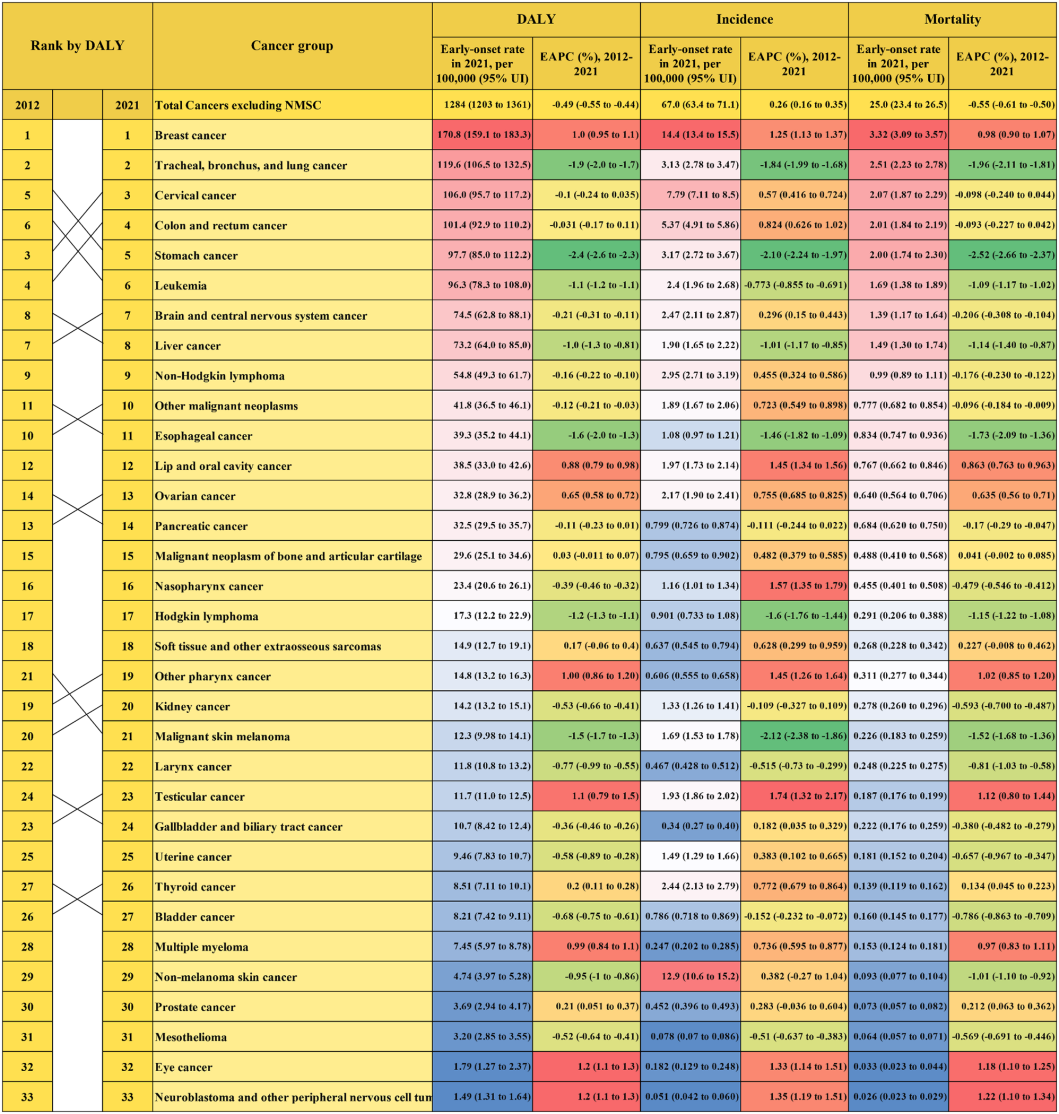
Global early-onset DALY, incidence and mortality rate in 2021 and their EAPCs among 2012–2021 for total cancers excluding NMSC and 33 cancer groups. (Note: DALY = disability-adjusted life years, EAPC = estimated annual percentage change, NMSC = non-melanoma skin cancer)

Globally, the incidence rate of new early-onset cancer (excluding NMSC) cases increased from 65.7 (95% UI, 63.6–67.9) per 100,000 in 2012 to 67.0 (95% UI, 63.4–71.1) per 100,000 in 2021, with an EAPC of 0.26% (0.16-0.35%), the EAPC was 0.57% (0.48-0.66%) among females while − 0.19% (-0.30% to -0.08%) among males (Fig. 1, Table S3).

Similarly, the number of early-onset cancer deaths increased from 8.25 million (95% UI, 7.77–8.61 million) in 2012 to 9.83 million (95% UI, 9.07–10.5 million) in 2021, an EAPC of 2.04% (95% CI, 1.98-2.11%). For early-onset mortality rate, the EAPC from 2012 to 2021 was − 0.55% (-0.61% to -0.50%).

The DALYs caused by total cancers increased from 220 million (95% UI, 211–229 million) in 2012 to 252 million (95% UI, 236–269 million), an EAPC of 1.59% (95% CI, 1.53-1.66%). For age-standardized DALY rate, the EAPC from 2012 to 2021 was − 0.82% (-0.87% to -0.76%). For early-onset DALY rate, the EAPC was − 0.49% (-0.55% to -0.44%).

10 cancer groups had an EAPC > 0 (*P* < 0.05), as eye cancer (1.2%, 1.1-1.3%), neuroblastoma and other peripheral nervous cell tumors (1.2%, 1.1-1.3%), testicular cancer (1.1%, 0.79-1.5%), and so on (Fig. 1, Table S3).

### Early-onset cancer burden by SDI

Early-onset cancer burden varied considerably across SDI quintiles in 2021 levels and rankings and trends during the 2012 to 2021. The following results exclude NMSC.

It is noteworthy that in the high SDI group, total cancers excluding NMSC were the sixth-most significant cause of DALYs among the 22 level 2 causes, while in the high-middle SDI group it was the fourth-ranked cause, and in the low SDI group, it was the fourteenth-ranked cause (Figure S1). A significant downward trend was observed in the DALY rates for multiple cancers in the high SDI region, while a rise was noted in the low-middle SDI and low SDI regions (Table S3).

**Fig. 2:**
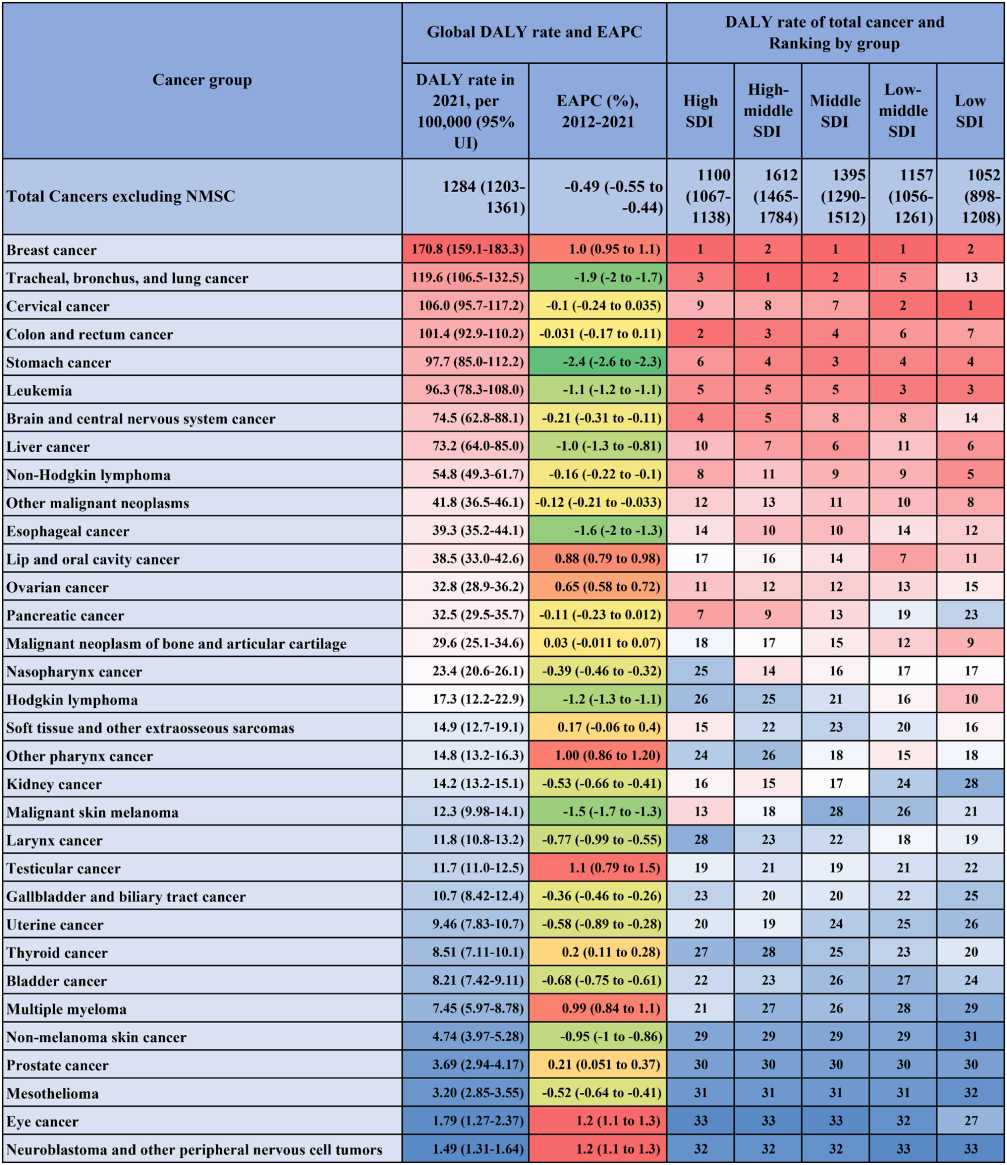
Global early-onset DALY rate in 2021 and the ranking by SDI for total cancers excluding NMSC and 33 cancer groups. (Note: DALY = disability-adjusted life years, EAPC = estimated annual percentage change, NMSC = non-melanoma skin cancer)

**Fig. 3:**
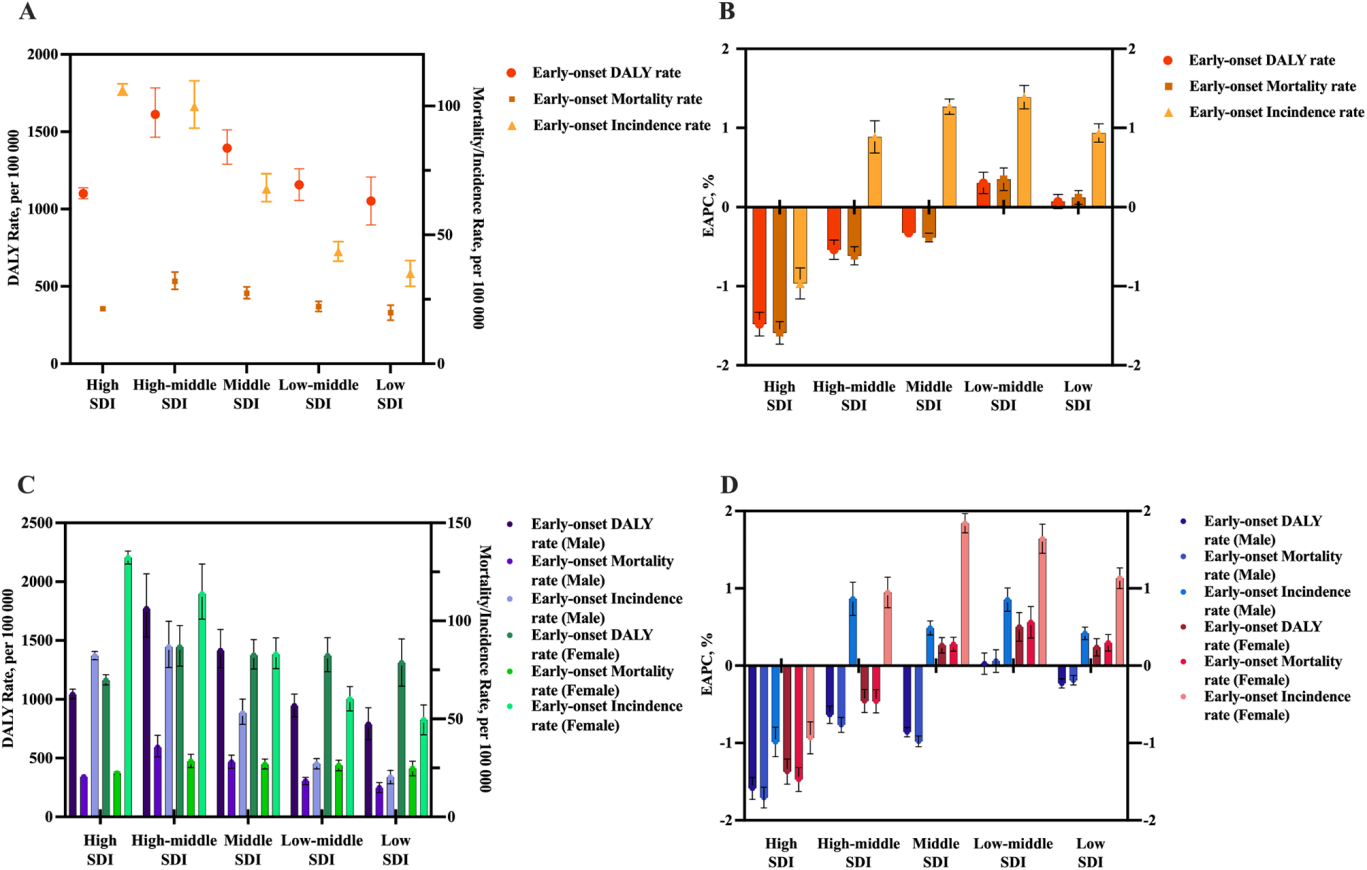
Early-onset rates in 2021 and their EAPCs among 2012–2021 of DALY, incidence, and death of total cancers excluding NMSC by SDI quintiles and genders. **A**: Early-onset rates in 2021 of DALY, incidence, and death of total cancers excluding NMSC by SDI quintiles. **B**: EAPC of early-onset rates among 2012–2021 of DALY, incidence, and death of total cancers excluding NMSC by SDI quintiles. **C**: Early-onset rates in 2021 of DALY, incidence, and death of total cancers excluding NMSC by SDI quintiles and genders. **D**: EAPC of early-onset rates among 2012–2021 of DALY, incidence, and death of total cancers excluding NMSC by SDI quintiles and genders

In the high SDI quintile in 2021, there were 1100 (95% UI, 1067–1138) DALYs per 100,000 estimated to be caused by total cancers excluding NMSC (Fig. 2). The most incidence rate were in the high SDI quintile. And the high SDI quintile had the highest absolute values of the negative EAPCs of early-onset DALY and mortality rate. The negative EAPC of age-standardized and early-onset incidence rate can be found only in the high SDI quintile, the same pattern is observed when analyzed by gender (Fig. 3D). The high-middle SDI had the highest early-onset rates of deaths and DALYs of all SDI quintiles. In low-middle SDI quintile, the upward trends of cancer mortality and incidence were significant in age-standardized and early-onset population. In middle, low-middle, and low SDI quintiles, the upward trends of DALYs, incidence and mortalities of early-onset cancers were more noteworthy in females (Fig. 3D).

**Fig. 4:**
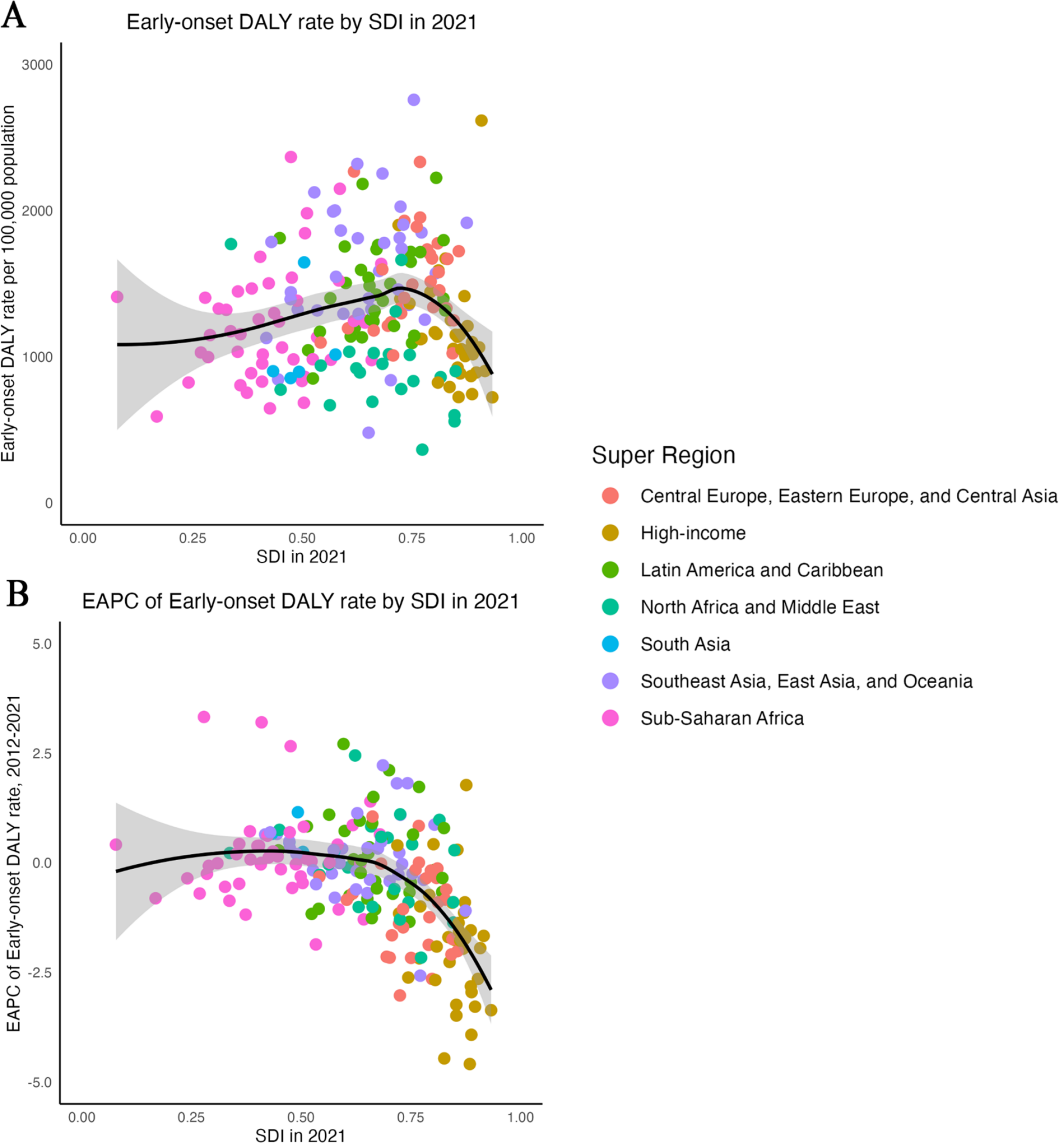
Early-onset DALY rate in 2021 and its EAPC among 2012–2021 of total cancer excluding NSMC for 204 countries and territories, by SDI (2021). **A**: Early-onset DALY rate in 2021 of total cancer excluding NSMC for 204 countries and territories by SDI. **B**: EAPC of early-onset DALY rate among 2012–2021 of total cancer excluding NSMC for 204 countries and territories by SDI

The LOESS model curves revealed that the early-onset DALY rate increases gradually as the SDI rises, reaching a peak before declining rapidly as the SDI continues to rise. With regard to the EAPC, it can be observed that when the SDI attains a specific elevated level, the EAPC assumes a negative value and diminishes in magnitude (Fig. 4).

## Discussion

The results of this systematic analysis highlight the increasing early-onset cancer burden globally, providing a reasonable estimate for the Disability-Adjusted Life Years (DALYs), incidence, and mortality of early-onset cancer. The primary findings are as follow: first, in people aged 15–49 years, DALYs caused by cancer ranked high among the 22 secondary diseases. Secondly, the incidence rate of early-onset cancers excluding NMSC is rising annually (EAPC = 0.26%, 95%CI: 0.16-0.35%) among 2012–2021, particularly in the other SDI quantiles except high quantile. Furthermore, the rates of DALY and mortality are also on the rise in low-middle and low SDI regions. Thirdly, some key early-onset cancers, variety among different SDI quintiles, and gender disparities were noteworthy. The findings are consistent with the trend previously reported in the literature [2, 22–25], the overall disease burden of early-onset cancers has continued to rise in recent years. Although advances in medical technology in recent decades have led to advances in the diagnosis and treatment of many cancers, there is still a need to further strengthen research and policy interventions for early-onset cancers [22, 26]. 

### Highlighted early-onset cancer groups

The highest DALY (171 per 100,000, 159–183), incidence rate, and mortality rate were observed for breast cancer among the early-onset cancer groups, with these metrics demonstrating a notable upward trend over the 10-year period. This is a cause for concern, given that the incidence rate of breast cancer is significantly higher than that of other cancers, even exceeding that of NMSC, which is highly incident but less fatal. Moreover, these figures are based on the overall population, indicating that the actual burden among women is likely to be approximately twice as high (DALY rate in female: 342/100,000). Similarly, early-onset cervical cancer has the third highest rate of DALY, incidence and mortality in the entire population aged 15–49 years. As a female-specific neoplasm, the DALY rate for cervical cancer is 2.5 times higher than that for TBL, the third leading cause of early-onset cancers in women. Ovarian cancer has the 13th highest disability-adjusted life year (DALY) rate among all population and the 7th highest among women, with all three rates (DALY, incidence, and mortality) exhibiting a significant upward trend and a high estimated annual percentage change (EAPC). More perspective research is urgently required to investigate the potential contributing factors behind the persistent rise in breast cancer incidence rates, and to develop effective strategies to mitigate this trend. This escalation may be linked to various environmental, dietary, and lifestyle-related determinants [8, 27]. Excessive work stress and psychological factors may also contribute to increased disease risk [27–31]. Furthermore, some studies have indicated that this phenomenon may be associated with factors such as changes in the lifestyle patterns of the population and environmental pollution [32–34]. 

The burden of both TBL and stomach cancer, as measured by DALY and mortality rates was high, while incidence rates were intermediate. Notably, a significant downward trend was observed in all three indicators across these cancer groups, which may be related to early screening and community interventions for lung cancer and stomach cancer [35, 36]. An investigative study [37] conducted in the United States has concluded that improvements in the treatment of lung cancer, reductions in exposure to second-hand smoke, and potentially reductions in exposure to other risk factors have likely contributed to the decline in non-smoking-related lung cancer mortality [38, 39]. Nevertheless, sustained public health initiatives are required to further diminish smoking prevalence and reduce disparities, particularly in communities where smoking prevalence remains elevated. Given the changing trends, eye cancer and neuroblastoma and other peripheral nervous cell tumors deserve attention because of their high increasing rates of DALYs, morbidity and mortality, albeit these rates are the lowest among all cancers. Ultraviolet radiation and autoimmune diseases like HIV or HPV infection are risk factors for eye cancer [40]. The growing burden of neuroblastoma may be linked to urban pollution, mining, and the release of carcinogens from factories [41]. 

### Focused regions by SDI

Overall, age-standardised mortality rates for cancer appear to be higher in more developed countries owing to aging and inappropriate lifestyles, according to previous studies and statements by the World Cancer Research Fund International. These risk factors are also becoming more prevalent in low- and middle-income countries [18, 42]. After segmenting the regions by SDI, we discover that the cancer with the highest DALY rate is TBL cancer in the high-middle region and cervical cancer in the low-SDI region. DALY rates of colon and rectal cancer were higher in high and high-middle SDI areas, with studies [22, 43] suggesting that aging and population growth play a major driving role. Pancreatic cancer ranked 7th and 9th in the high and high-middle SDI regions, but ranked 23rd in the low SDI region, which may be related to dietary habits, etc., and some studies [44, 45] have supported that a certain percentage of pancreatic cancer DALYs can be attributed to known risk factors: smoking (13.3%), high BMI (5.6%), and high fasting glucose (3.2%). Some scholars have observed and conducted comprehensive analyses, indicating that there are variations in the burden of female breast cancer (FBC) across the globe. These findings highlight the necessity for greater attention to be directed towards the control of regions with medium and low SDI. It is imperative that public health and tumor control experts devote more attention to areas and populations at high risk of FBC, priorities prevention and rehabilitation, and undertake further epidemiological studies to elucidate the risk factors underlying the rising incidence of FBC in areas and populations at high risk [46]. 

Malignant tumors, including those of the lung, liver, colorectal, esophageal, breast and gastric cancers, not only represent a significant health risk for local populations but also impose considerable economic costs [47]. It is imperative to develop screening strategies for early-stage disease that are simple to implement and widely accepted, as this will lead to significant savings in health expenditure [48, 49]. Furthermore, the fairness and affordability of cancer treatment are becoming increasingly contentious from both individual and societal perspectives. The survival rates observed in high-income countries vary depending on a number of factors, including the level of education attained by the population, the availability of specialist care, the efficacy of the treatment provided and the insurance status of the patient [50]. The complete potential of cancer prevention in reducing both the incidence and mortality rates remains unfulfilled. Efforts in low-income countries exhibit a notable lag behind those in other regions [50, 51]. The accessibility of cancer treatment is contingent upon a complex interplay of gender and a multitude of socio-environmental factors. Although gender is a significant factor, it is influenced by a multitude of socio-environmental factors [52]. Future research should concentrate on the creation of targeted interventions to address these complex barriers and to facilitate equal access to healthcare for demographic groups from all over the world. Furthermore, this study defines the age threshold for early-onset cancer as ≤ 50 years to maintain consistency with global comparative frameworks and align with established epidemiological research paradigms on premature malignancies. However, significant variations in national age structures warrant careful consideration. Notably, despite possessing younger population demographics, certain developing nations exhibit even earlier cancer manifestation patterns. For instance, comparative analyses reveal that the median age of cancer onset in India occurs approximately 10 years earlier than in Western populations [53]. This epidemiological disparity underscores the imperative for policymakers at both national and international levels to develop context-specific intervention strategies that synergize evidence-based research with localized demographic characteristics when formulating cancer control policies.

### Strengths and limitations of this study

The findings of this study highlight the significant burden of multiple cancer groups among individuals aged 15–49 years old. Furthermore, we explore the association of cancer burden and its trends over the last decade with the SDI, and provide an immediate update on the dynamics of early-onset cancers. By conducting a comprehensive temporal analysis over a decade (2012–2021), the study provides valuable insights into long-term trends and the effectiveness of public health interventions. The global scope of the research allows for the identification of the types of early-onset cancers that should be of greatest concern, as well as their severity. However, our study has several potential limitations. Firstly, the GBD database is dependent upon nationally reported data and predictive modelling, which may not be wholly representative of the actual situation, particularly in regions where data are scarce or of poor quality [18]. However, the GBD collaborators have addressed the underreporting and comparability by integrating multiple data sources and applying methods such as expansion factors, estimation of asymptomatic infections, and combinatorial mathematical models in their modeling strategy, aiming to accurately reflect the epidemiological characteristics [19, 54]. Secondly, variations in testing standards and testing accuracy across countries and regions may affect the consistency of results. Thirdly, this paper presents a systematic study and is therefore unable to explore the factors and causal relationships associated with the various cancer burdens. Fourth, the age threshold definition for early-onset cancer may not be applicable to all countries. Therefore, policy formulation requires tailored modifications aligned with national epidemiological profiles to ensure context-specific intervention efficacy.

## Conclusion

The issue of early-onset cancers represents a growing concern, with the burden and EAPC of such diseases related to the level of SDI. It is imperative to priorities the alleviation of gender inequalities in cancer and the intensified burden of cancer in underdeveloped regions. This necessitates the formulation and implementation of efficacious early screening strategies for an array of cancers, the investigation of multimorbidity mechanisms, and the utilization of the World Health Organization’s capabilities to reinforce the provision of screening resources in disadvantaged areas.

## Electronic supplementary material

Below is the link to the electronic supplementary material.

Supplementary Material 1

## Data Availability

No datasets were generated or analysed during the current study.

## Declarations

### Copyright and license agreement

All authors of the manuscript have read and agreed to its content and are accountable for all aspects of the accuracy and integrity of the manuscript in accordance with ICMJE criteria.
