# Supplementary material for: Disparities in the incidence, mortality and disability-adjusted life years of 33 early-onset cancer groups globally, 2012–2021: a systematic analysis
Source: Exp Hematol Oncol. 2025 Mar 17;14:38. doi: 10.1186/s40164-025-00634-7 (PMC11912769; doi:10.1186/s40164-025-00634-7)
Supplement: Supplementary file 1 — Supplementary Material 1 [file 40164_2025_634_MOESM1_ESM.docx]

# Supplementary Materials

**Disparities in the incidence, mortality and disability-adjusted life years of 33 early-onset cancer groups globally, 2012-2021: a systematic analysis**

**Contents**

[Supplementary Materials 0](#_Toc180548300)

[Appendix 2](#_Toc180548301)

[Appendix 1. List of International Classification of Diseases (ICD) codes mapped to 34 cancer groups in GBD 2021 2](#_Toc180548302)

[Appendix 2. GBD data collection, modeling/analysis, and dissemination 7](#_Toc180548303)

[Table S1. Classification of countries and territories by 7 global burden of disease (GBD) super regions and 21 GBD regions 8](#_Toc180548304)

[Table S2 Global burden of cancers by age grouping, measure, sex, and matrix in 2021. 9](#_Toc180548305)

[Figure S1. ﻿Ranking of total cancer absolute disability-adjusted life years (DALYs) in 2021 among the 22 level 2 categories of disease in the Global Burden of Disease (GBD) by quintile of sociodemographic index (SDI) 47](#_Toc180548306)

### Appendix

### Appendix 1. List of International Classification of Diseases (ICD) codes mapped to 34 cancer groups in GBD 2021

| **ICD codes mapped to non-fatal causes and injuries in the GBD 2021** | | | | | | | **ICD codes mapped to the causes of death** | |
| --- | --- | --- | --- | --- | --- | --- | --- | --- |
| Cause ID | Cause Hierarchy Level | Cause Name | ICD10 | ICD10 Used in Hospital/Claims Analyses | ICD9 | ICD9 Used in Hospital/Claims Analyses | ICD10 | ICD9 |
| 410 | 2 | Neoplasms | C00-C45.9, C46.6, C47-C79.9, C8-D24.9, D26.0-D39.9, D4-D49.9, D54, E34.0, K62.0-K62.3, K63.5, N60-N60.99, N84.0-N84.1, N87-N87.9 | C44.01-D24.9, D26.0-D49.9, E34.0, K62.0-K62.1, K63.5, N60-N60.99, N84.0-N84.1, N87-N87.9 | 140-175.9, 177-217.8, 219-237.6, 237.70-237.72, 237.9-239.9, 569.0, 610-610.9, 622.1-622.2, 622.7, V07.39, V10-V11, V13.22-V13.24, V16-V16.9, V42.4, V42.81-V42.82, V59.2-V59.3, V72.32, V76-V76.9 | 173.01-217.8, 219-237.6, 237.70-237.72, 237.9-239.9, 569.0, 610-610.9, 622.1-622.2, 622.7 | C00-C13.9, C15-C22.8, C23-C25.9, C30-C34.9, C37-C38.8, C40-C41.9, C43-C45.9, C47-C54.9, C56-C57.8, C60-C63.8, C64-C67.9, C68.0-C68.8, C69.0-C69.8, C70-C73.9, C75-C75.8, C81-C82.9, C83.0-C83.8, C84-C85.0, C85.2-C85.8, C86-C86.6, C88-C91.0, C91.2-C91.3, C91.6, C92-C92.6, C93-C93.1, C93.3, C93.8, C94-C94.5, C94.7-C96.9, D00.1-D00.2, D01.0-D01.3, D02.0-D02.3, D03-D06.9, D07.0-D07.2, D07.4-D07.5, D09.0, D09.2-D09.3, D09.8, D10.0-D10.7, D11-D12.9, D13.0-D13.7, D14.0-D14.3, D15-D16.9, D22-D24.9, D26.0-D27.9, D28.0-D28.1, D28.7, D29.0-D29.8, D30.0-D30.8, D31-D36, D36.1-D36.7, D37.1-D37.5, D38.0-D38.5, D39.1-D39.2, D39.8, D40.0-D40.8, D41.0-D41.8, D42-D43.9, D44.0-D44.8, D45-D47.9, D48.0-D48.6, D49.2-D49.4, D49.6, K62.0-K62.1, K63.5, N60-N60.9, N84.0-N84.1, N87-N87.9 | 140-148.9, 150-155.1, 155.3-158.9, 160-164.9, 170-175.9, 180-180.9, 182-183.8, 184.0-184.4, 184.8, 185-186.9, 187.1-187.8, 188-188.9, 189.0-189.8, 190-190.8, 191-193.9, 194.1-194.8, 200-202.8, 203-204.0, 204.2, 205-205.3, 206-206.1, 207-208.9, 209.0-209.1, 209.4-209.5, 210.0-210.9, 211.0-211.8, 212.0-212.8, 213-213.9, 217-217.8, 219.0, 220-220.9, 221.0-221.8, 222.0-222.8, 223.0-223.8, 224-228.9, 229.0, 229.8, 230.1-230.8, 231.0-231.2, 232-232.9, 233.0-233.2, 233.4-233.5, 233.7, 234.0-234.8, 235.0, 235.4, 235.6-235.8, 236.1-236.2, 236.4-236.5, 236.7, 237-237.3, 237.5-237.9, 238.0-238.9, 239.2-239.4, 239.6, 569.0, 610-610.9, 622.1-622.2, 622.7 |
| 444 | 3 | Lip and oral cavity cancer | C00-C07, C08-C08.9 |  | 140-145.9, V76.42 |  | C00-C08.9, D10.0-D10.5, D11-D11.9 | 140-145.9, 210.0-210.6, 235.0 |
| 447 | 3 | Nasopharynx cancer | C11-C11.9 |  | 147-147.9 |  | C11-C11.9, D10.6 | 147-147.9, 210.7-210.9 |
| 450 | 3 | Other pharynx cancer | C09-C10.9, C12-C13.9 |  | 146-146.9, 148-148.9 |  | C09-C10.9, C12-C13.9, D10.7 | 146-146.9, 148-148.9 |
| 411 | 3 | Esophageal cancer | C15-C15.9 |  | 150-150.9 |  | C15-C15.9, D00.1, D13.0 | 150-150.9, 211.0, 230.1 |
| 414 | 3 | Stomach cancer | C16-C16.9 |  | 151-151.9, 209.23, V10.04 |  | C16-C16.9, D00.2, D13.1, D37.1 | 151-151.9, 211.1, 230.2 |
| 441 | 3 | Colon and rectum cancer | C18-C19.0, C20, C21-C21.8 |  | 153-154.9, 209.1-209.17, V10.05-V10.06, V76.41, V76.5-V76.52 |  | C18-C21.9, D01.0-D01.3, D12-D12.9, D37.3-D37.5 | 153-154.9, 209.1, 209.5, 211.3-211.4, 230.3-230.6, 569.0 |
| 417 | 3 | Liver cancer | C22-C22.4, C22.7-C22.8 |  | 155-155.9, V10.07 |  | C22-C22.8, D13.4 | 155-155.1, 155.3-155.9, 211.5 |
| 453 | 3 | Gallbladder and biliary tract cancer | C23, C24-C24.9 |  | 156-156.9 |  | C23-C24.9, D13.5 | 156-156.9 |
| 456 | 3 | Pancreatic cancer | C25-C25.9 |  | 157-157.9 |  | C25-C25.9, D13.6-D13.7 | 157-157.9, 211.6-211.7 |
| 423 | 3 | Larynx cancer | C32-C32.9 |  | 161-161.9, V10.21 |  | C32-C32.9, D02.0, D14.1, D38.0 | 161-161.9, 212.1, 231.0, 235.6 |
| 426 | 3 | Tracheal, bronchus, and lung cancer | C33, C34-C34.92 |  | 162-162.9, 209.21, V10.1-V10.20, V16.1-V16.2, V16.4-V16.40 |  | C33-C34.9, D02.1-D02.3, D14.2-D14.3, D38.1 | 162-162.9, 212.2-212.3, 231.1-231.2, 235.7 |
| 459 | 3 | Malignant skin melanoma | C43-C43.9 |  | 172-172.9 |  | C43-C43.9, D03-D03.9, D22-D23.9, D48.5 | 172-172.9 |
| 462 | 3 | Non-melanoma skin cancer | C44.01-C44.99 | C44.01-C44.92 | 173-173.99 | 173.01-173.92 | C44-C44.9, D04-D04.9, D49.2 | 173-173.9, 222.4, 232-232.9, 238.2 |
| 1011 | 3 | Soft tissue and other extraosseous sarcomas | C49-C49.9 |  | 171-171.9 |  | C49-C49.9 | 171-171.9 |
| 1012 | 3 | Malignant neoplasm of bone and articular cartilage | C40-C40.92, C41.0-C41.4, C41.8-C41.9 |  | 170-170.9 |  | C40-C41.9 | 170-170.9 |
| 429 | 3 | Breast cancer | C50-C50.629, C50.8-C50.929 |  | 174-175.9, V10.3, V16.3 |  | C50-C50.9, D05-D05.9, D24-D24.9, D48.6, D49.3 | 174-175.9, 217-217.8, 233.0, 238.3, 239.3, 610-610.9 |
| 432 | 3 | Cervical cancer | C53-C53.9 |  | 180-180.9, V10.41, V72.32 |  | C53-C53.9, D06-D06.9, D26.0 | 180-180.9, 219.0, 233.1, 622.1-622.2, 622.7 |
| 435 | 3 | Uterine cancer | C54-C54.3, C54.8-C54.9 |  | 182-182.9 |  | C54-C54.9, D07.0-D07.2, D26.1-D26.9 | 182-182.9, 233.2 |
| 465 | 3 | Ovarian cancer | C56-C56.2, C56.9 |  | 183-183.0, 183.8-183.9, V10.43, V16.41 |  | C56-C56.9, D27-D27.9, D39.1 | 183-183.0, 220-220.9, 236.2 |
| 438 | 3 | Prostate cancer | C61-C61.9 |  | 185-185.9, V10.46, V16.42, V76.44 |  | C61-C61.9, D07.5, D29.1, D40.0 | 185-185.9, 222.2, 236.5 |
| 468 | 3 | Testicular cancer | C62-C62.92 |  | 186-186.9, V10.47-V10.48, V16.43 |  | C62-C62.9, D29.2-D29.8, D40.1-D40.8 | 186-186.9, 222.0, 222.3, 236.4 |
| 471 | 3 | Kidney cancer | C64-C64.2, C64.9-C65.9 |  | 189-189.1, 189.5-189.6, 209.24 |  | C64-C65.9, D30.0-D30.1, D41.0-D41.1 | 189.0-189.1, 189.5-189.6, 223.0-223.1 |
| 474 | 3 | Bladder cancer | C67-C67.9 |  | 188-188.9, V10.51, V16.52, V76.3 |  | C67-C67.9, D09.0, D30.3, D41.4-D41.8, D49.4 | 188-188.9, 223.3, 233.7, 236.7, 239.4 |
| 477 | 3 | Brain and central nervous system cancer | C70-C70.1, C70.9-C72.9 |  | 191-191.9 |  | C70-C72.9, C75.1-C75.3 | 191-192.9, 194.3-194.4 |
| 1008 | 3 | Eye cancer | C69-C69.92 |  | 190-190.9 |  | C69.0-C69.8 | 190-190.8 |
| 1013 | 3 | Neuroblastoma and other peripheral nervous cell tumors | C47-C47.9 |  |  |  | C47-C47.9 |  |
| 480 | 3 | Thyroid cancer | C73 |  | 193-193.9 |  | C73-C73.9, D09.3, D09.8, D34-D34.9, D44.0 | 193-193.9, 226-226.9 |
| 483 | 3 | Mesothelioma | C45-C45.2, C45.7, C45.9 |  |  |  | C45-C45.9 |  |
| 484 | 3 | Hodgkin lymphoma | C81-C81.49, C81.7-C81.79, C81.9-C81.99 |  | 201-201.98, V10.72 |  | C81-C81.9 | 201-201.9 |
| 485 | 3 | Non-Hodgkin lymphoma | C82-C85.29, C85.7-C86.6, C96-C96.9 |  | 200-200.9, 202-202.98 |  | C82-C82.9, C83.0-C83.8, C84-C85.0, C85.2-C85.8, C86-C86.6, C96-C96.9 | 200-200.9, 202-202.8 |
| 486 | 3 | Multiple myeloma | C88-C90.32 |  | 203-203.9 |  | C88-C90.9 | 203-203.9 |
| 487 | 3 | Leukemia | C91-C93.7, C93.9-C95.2, C95.7-C95.92 |  | 204-208.92, V10.59-V10.69, V16.6 |  | C91-C91.0, C91.2-C91.3, C91.6, C92-C92.6, C93-C93.1, C93.3, C93.8, C94-C94.5, C94.7-C95.9 | 204-204.0, 204.2, 205-205.3, 206-206.1, 207-208.9 |
| 489 | 3 | Other malignant neoplasms | C17-C17.9, C30-C30.1, C31-C31.9, C37-C37.0, C38-C38.8, C41, C44-C44.00, C48-C48.9, C4A, C51-C52, C57-C57.8, C58-C58.0, C60-C60.9, C63-C63.8, C66-C66.9, C68.0-C68.8, C74-C75.5, C75.8 |  | 152-152.9, 158-158.9, 160-160.9, 163-164.9, 181-181.9, 183.2-183.5, 184-184.9, 187-187.9, 189.2-189.4, 189.8-189.9, 192-192.9, 194.1-194.8, 209-209.03, 209.22, 209.25-209.27, 209.31-209.36 |  | C17-C17.9, C30-C31.9, C37-C38.8, C48-C48.9, C4A, C51-C52.9, C57-C57.8, C60-C60.9, C63-C63.8, C66-C66.9, C68.0-C68.8, C75-C75.0, C75.4-C75.8, D07.4, D09.2, D13.2-D13.3, D14.0, D15-D16.9, D28.0-D28.1, D28.7, D29.0, D30.2, D30.4-D30.8, D31-D31.9, D35-D35.2, D35.5-D36, D36.1-D36.7, D37.2, D38.2-D38.5, D39.2, D39.8, D41.2-D41.3, D44.1-D44.8, D48.0-D48.4 | 152-152.9, 158-158.9, 160-160.9, 163-164.9, 183.2-183.8, 184.0-184.4, 184.8, 187.1-187.8, 189.2-189.4, 189.8, 194.1, 194.5-194.8, 209.0, 209.4, 211.2, 211.8, 212.0, 212.4-212.8, 213-213.9, 221.0-221.8, 222.1, 222.8, 223.2, 223.8, 224-224.9, 227-228.9, 229.0, 229.8, 230.7-230.8, 233.4-233.5, 234.0-234.8, 235.4, 235.8, 236.1, 238.0-238.1, 239.2 |
| 490 | 3 | Other neoplasms | C75.90-C75.92, D00-D24.9, D26.0-D39.9, D4-D49.9, E34.0, K62.0-K62.3, K63.5, N60-N60.99, N84.0-N84.1, N87-N87.9 | D00-D24.9, D26.0-D49.9, E34.0, K62.0-K62.1, K63.5, N60-N60.99, N84.0-N84.1, N87-N87.9 | 209.4-209.57, 209.61, 209.63-209.67, 210.0-217.8, 219-237.6, 237.70-237.72, 237.9-239.9, 569.0, 610-610.9, 622.1-622.2, 622.7 | 209.4-217.8, 219-237.6, 237.70-237.72, 237.9-239.9, 569.0, 610-610.9, 622.1-622.2, 622.7 | D32-D33.9, D35.3-D35.4, D42-D43.9, D45-D47.9, D49.6, K62.0-K62.1, K63.5, N60-N60.9, N84.0-N84.1, N87-N87.9 | 225-225.9, 237-237.3, 237.5-237.9, 238.4-238.9, 239.6 |

### Appendix 2. GBD data collection, modeling/analysis, and dissemination

The Global Burden of Disease (GBD) study is a comprehensive research initiative aimed at systematically assessing the health status and disease burden of populations worldwide. An international network comprising over 11,500 collaborators from 164 countries and territories contributed to the generation of GBD metrics through data provision, review, and analysis. GBD data collection involves diverse sources, including epidemiological surveys, hospital records, vital registration systems, disease surveillance systems, and additional sources such as academic papers and policy reports (https://ghdx.healthdata.org/gbd-2021/sources). The data is standardized using the International Classification of Diseases (ICD) codes to ensure accuracy and comparability (https://ghdx.healthdata.org/record/ihme-data/gbd-2021-cause-icd-code-mappings). Sophisticated modeling tools, such as DisMod-MR and Spatiotemporal Gaussian Process Regression (ST-GPR), are employed to estimate prevalence, incidence, and mortality rates. Data processing includes corrections for heterogeneity and biases, as well as uncertainty analysis through Monte Carlo simulations. Key health metrics used are Disability-Adjusted Life Years (DALYs), Years of Life Lost (YLLs), and Years Lived with Disability (YLDs) (https://www.healthdata.org/gbd/methods-appendices-2021/cancers). Dissemination of GBD findings is achieved through scientific publications (https://www.healthdata.org/research-analysis/gbd-publications), and interactive tools like GBD Compare and Viz Hub (https://www.healthdata.org/research-analysis/gbd-data). These tools facilitate the exploration and comparison of health data across regions and time periods. The primary goal of GBD findings is to provide a comprehensive framework for understanding global and local health trends, thereby supporting evidence-based health decision-making and resource allocation.

### Table S1. Classification of countries and territories by 7 global burden of disease (GBD) super regions and 21 GBD regions

| **GBD Super Region** | **GBD Region** | **Countries and Territories** |
| --- | --- | --- |
| Central Europe, Eastern Europe, and Central Asia | Central Asia | Armenia, Azerbaijan, Georgia, Kazakhstan, Kyrgyzstan, Mongolia, Tajikistan, Turkmenistan, Uzbekistan |
|  | Central Europe | Albania, Bosnia and Herzegovina, Bulgaria, Croatia, Czechia, Hungary, Montenegro, North Macedonia, Poland, Romania, Serbia, Slovakia, Slovenia |
|  | Eastern Europe | Belarus, Estonia, Latvia, Lithuania, Republic of Moldova, Ukraine, Russian Federation |
| High-income | Australasia | Australia, New Zealand |
|  | High-income Asia Pacific | Japan, South Korea, Singapore, Brunei Darussalam |
|  | Southern Latin America | Argentina, Chile, Uruguay |
|  | Western Europe | Andorra, Austria, Belgium, Cyprus, Denmark, Finland, France, Germany, Greece, Iceland, Ireland, Italy, Israel, Luxembourg, Malta, Monaco, Netherlands, Norway, Portugal, San Marino, Spain, Sweden, Switzerland, United Kingdom. |
|  | High-income North America | Canada, Greenland, United States of America |
| Latin America and Caribbean | Caribbean | Antigua and Barbuda, Bahamas, Barbados, Belize, Bermuda, Cuba, Dominica, Dominican Republic, Grenada, Guyana, Haiti, Jamaica, Puerto Rico, Saint Kitts and Nevis, Saint Lucia, Saint Vincent and the Grenadines, Suriname, Trinidad and Tobago, United states Virgin Islands |
|  | Andean Latin America | Bolivia, Ecuador, Peru |
|  | Central Latin America | Colombia, Costa Rica, El Salvador, Guatemala, Honduras, Mexico, Nicaragua, Panama, Venezuela |
|  | Tropical Latin America | Brazil, Paraguay |
| North Africa and Middle East | North Africa and Middle East | Afghanistan, Algeria, Bahrain, Egypt, Iran, Iraq, Jordan, Kuwait, Lebanon, Libya, Morocco, Oman, Palestine, Qatar, Saudi Arabia, Sudan, Syrian Arab Republic, Tunisia, Tükiye, United Arab Emirates, Yemen |
| South Asia | South Asia | Bangladesh, Bhutan, India, Nepal, Pakistan |
| Southeast Asia, East Asia, and Oceania | East Asia | China, Democratic People’s Republic of Korea, Taiwan (Province of China) |
|  | Southeast Asia | Cambodia, Indonesia, Laos, Malaysia, Maldives, Mauritius, Myanmar, Philippines, Seychelles, Sri Lanka, Thailand, Timor-Leste, Viet Nam. |
|  | Oceania | American Samoa, Cook Islands, Fiji, Guam, Kiribati, Marshall Islands, Micronesia, Nauru, Niue, Northern Mariana Islands, Palau, Papua New Guinea, Samoa, Solomon Islands, Tokelau, Tonga, Tuvalu, Vanuatu. |
| Sub-Saharan Africa | Western Sub-Saharan Africa | Benin, Burkina Faso, Cape Verde, Cameroon, Chad, Cote d'lvoire, Gambia, Ghana, Guinea, Guinea-Bissau, Liberia, Mali, Mauritania, Niger, Nigeria, Sao Tome and Principe, Senegal, Sierra Leone, Togo |
|  | Central Sub-Saharan Africa | Angola, Cameroon, Central African Republic, Congo, Democratic Republic of the Congo, Equatorial Guinea, Gabon |
|  | Eastern Sub-Saharan Africa | Burundi, Comoros, Djibouti, Eritrea, Ethiopia, Kenya, Madagascar, Malawi, Mozambique, Rwanda, Somalia, South Sudan, Uganda, United Republic of Tanzania, Zambia. |
|  | Southern Sub-Saharan Africa | Botswana, Eswatini, Lesotho, Namibia, South Africa, Zimbabwe. |

### Table S2 Global burden of cancers by age grouping, measure, sex, and matrix in 2021.

| **Cause** | **Age grouping** | **Measure** | **Sex** | **Number (95% UI)** | **Rate (per 100,000, 95% UI)** |
| --- | --- | --- | --- | --- | --- |
| Neoplasms | All ages | Deaths | Male | 56.01 (51.73, 61.31) | 141.5 (130.6, 154.8) |
|  |  |  | Female | 42.87 (38.88, 46.24) | 109 (98.88, 117.6) |
|  |  |  | Both | 98.88 (91.25, 105.9) | 125.3 (115.6, 134.1) |
|  |  | DALYs (Disability-Adjusted Life Years) | Male | 1417 (1303, 1552) | 3578 (3291, 3921) |
|  |  |  | Female | 1116 (1036, 1197) | 2839 (2634, 3043) |
|  |  |  | Both | 2533 (2375, 2706) | 3210 (3009, 3429) |
|  |  | Incidence | Male | 270.6 (243.3, 300) | 683.5 (614.5, 757.6) |
|  |  |  | Female | 394.2 (338.7, 454.1) | 1002 (861.5, 1155) |
|  |  |  | Both | 664.8 (583.4, 749.8) | 842.4 (739.2, 950.2) |
|  | 15-49 years | Deaths | Male | 4.822 (4.4, 5.29) | 24.11 (22, 26.45) |
|  |  |  | Female | 5.094 (4.715, 5.489) | 26.14 (24.19, 28.17) |
|  |  |  | Both | 9.916 (9.296, 10.53) | 25.11 (23.54, 26.66) |
|  |  | DALYs (Disability-Adjusted Life Years) | Male | 245.8 (225, 269.1) | 1229 (1125, 1346) |
|  |  |  | Female | 264.3 (244.3, 284.9) | 1356 (1253, 1462) |
|  |  |  | Both | 510.1 (478.2, 540.8) | 1292 (1211, 1370) |
|  |  | Incidence | Male | 65.46 (53.22, 80.72) | 327.3 (266.2, 403.7) |
|  |  |  | Female | 170.6 (133.5, 211.8) | 875.6 (685.3, 1087) |
|  |  |  | Both | 236.1 (186.4, 292.4) | 598 (472.1, 740.6) |
|  | Age-standardized | Deaths | Male |  | 145.7 (134.5, 158.7) |
|  |  |  | Female |  | 93.6 (85.02, 100.9) |
|  |  |  | Both |  | 116.5 (107.3, 124.7) |
|  |  | DALYs (Disability-Adjusted Life Years) | Male |  | 3485 (3209, 3815) |
|  |  |  | Female |  | 2508 (2327, 2687) |
|  |  |  | Both |  | 2954 (2769, 3154) |
|  |  | Incidence | Male |  | 673.1 (608.1, 742.3) |
|  |  |  | Female |  | 923.4 (790.5, 1073) |
|  |  |  | Both |  | 790.3 (694.4, 893) |
| Total cancers | All ages | Deaths | Male | 55.7 (51.4, 60.96) | 140.7 (129.8, 154) |
|  |  |  | Female | 42.63 (38.66, 45.98) | 108.4 (98.33, 117) |
|  |  |  | Both | 98.33 (90.75, 105.3) | 124.6 (115, 133.4) |
|  |  | DALYs (Disability-Adjusted Life Years) | Male | 1410 (1296, 1545) | 3560 (3273, 3902) |
|  |  |  | Female | 1111 (1031, 1191) | 2826 (2622, 3030) |
|  |  |  | Both | 2521 (2363, 2693) | 3195 (2995, 3413) |
|  |  | Incidence | Male | 128 (120.2, 135.5) | 323.2 (303.6, 342.3) |
|  |  |  | Female | 107.7 (100.4, 114.4) | 273.9 (255.4, 290.9) |
|  |  |  | Both | 235.7 (222, 248.5) | 298.6 (281.3, 314.9) |
|  | 15-49 years | Deaths | Male | 4.811 (4.39, 5.278) | 24.06 (21.95, 26.39) |
|  |  |  | Female | 5.086 (4.707, 5.48) | 26.1 (24.15, 28.12) |
|  |  |  | Both | 9.896 (9.278, 10.51) | 25.06 (23.5, 26.61) |
|  |  | DALYs (Disability-Adjusted Life Years) | Male | 245 (224.3, 268.2) | 1225 (1122, 1341) |
|  |  |  | Female | 263.7 (243.6, 284.3) | 1353 (1250, 1459) |
|  |  |  | Both | 508.7 (476.8, 539.6) | 1288 (1208, 1366) |
|  |  | Incidence | Male | 12.51 (11.63, 13.6) | 62.54 (58.16, 68.02) |
|  |  |  | Female | 19.05 (17.77, 20.37) | 97.73 (91.18, 104.5) |
|  |  |  | Both | 31.55 (29.82, 33.4) | 79.91 (75.52, 84.6) |
|  | Age-standardized | Deaths | Male |  | 144.8 (133.7, 157.8) |
|  |  |  | Female |  | 93.08 (84.61, 100.4) |
|  |  |  | Both |  | 115.8 (106.7, 124) |
|  |  | DALYs (Disability-Adjusted Life Years) | Male |  | 3466 (3192, 3794) |
|  |  |  | Female |  | 2496 (2317, 2675) |
|  |  |  | Both |  | 2939 (2755, 3139) |
|  |  | Incidence | Male |  | 325.3 (306.1, 343.9) |
|  |  |  | Female |  | 237.4 (221.8, 252.2) |
|  |  |  | Both |  | 275.2 (259, 290) |
| Total Cancers excluding Non-melanoma skin cancer | All ages | Deaths | Male | 55.38 (51.09, 60.62) | 139.9 (129, 153.1) |
|  |  |  | Female | 42.38 (38.44, 45.7) | 107.8 (97.77, 116.2) |
|  |  |  | Both | 97.76 (90.22, 104.7) | 123.9 (114.3, 132.7) |
|  |  | DALYs (Disability-Adjusted Life Years) | Male | 1402 (1289, 1537) | 3542 (3255, 3883) |
|  |  |  | Female | 1106 (1027, 1186) | 2814 (2611, 3016) |
|  |  |  | Both | 2509 (2352, 2681) | 3179 (2980, 3398) |
|  |  | Incidence | Male | 91.01 (84.6, 98.83) | 229.9 (213.7, 249.6) |
|  |  |  | Female | 81.28 (74.73, 87.46) | 206.7 (190.1, 222.4) |
|  |  |  | Both | 172.3 (160.8, 183.3) | 218.3 (203.8, 232.3) |
|  | 15-49 years | Deaths | Male | 4.79 (4.37, 5.255) | 23.95 (21.85, 26.28) |
|  |  |  | Female | 5.07 (4.692, 5.464) | 26.02 (24.08, 28.04) |
|  |  |  | Both | 9.86 (9.242, 10.47) | 24.97 (23.41, 26.51) |
|  |  | DALYs (Disability-Adjusted Life Years) | Male | 243.9 (223.2, 267) | 1220 (1116, 1335) |
|  |  |  | Female | 262.9 (242.9, 283.4) | 1349 (1246, 1454) |
|  |  |  | Both | 506.8 (474.9, 537.5) | 1284 (1203, 1361) |
|  |  | Incidence | Male | 10.24 (9.424, 11.16) | 51.19 (47.12, 55.83) |
|  |  |  | Female | 16.24 (15.12, 17.48) | 83.33 (77.58, 89.69) |
|  |  |  | Both | 26.47 (25.02, 28.07) | 67.05 (63.36, 71.09) |
|  | Age-standardized | Deaths | Male |  | 143.8 (132.9, 156.9) |
|  |  |  | Female |  | 92.55 (84.15, 99.78) |
|  |  |  | Both |  | 115.1 (106, 123.2) |
|  |  | DALYs (Disability-Adjusted Life Years) | Male |  | 3447 (3174, 3774) |
|  |  |  | Female |  | 2485 (2307, 2663) |
|  |  |  | Both |  | 2925 (2742, 3125) |
|  |  | Incidence | Male |  | 229.5 (213.6, 248.6) |
|  |  |  | Female |  | 180.1 (165.9, 193.7) |
|  |  |  | Both |  | 201.1 (187.4, 213.7) |

**Table S3. EAPC of DALY, mortality, incidence rate of early-onset cancer by SDI quintiles, sex, and 33 cancer groups.**

| **Global and SDI quintiles** | **Sex** | **Cancer group** | **EAPC of DALY rate (95% CI)** | **EAPC of mortality rate (95% CI)** | **EAPC of incidence rate (95% CI)** |
| --- | --- | --- | --- | --- | --- |
| Global | Both | Bladder cancer | -0.68 (-0.75, -0.61) | -0.79 (-0.86, -0.71) | -0.15 (-0.23, -0.072) |
| Global | Both | Brain and central nervous system cancer | -0.21 (-0.31, -0.11) | -0.21 (-0.31, -0.1) | 0.3 (0.15, 0.44) |
| Global | Both | Breast cancer | 1 (0.95, 1.1) | 0.98 (0.9, 1.1) | 1.2 (1.1, 1.4) |
| Global | Both | Cervical cancer | -0.1 (-0.24, 0.035) | -0.098 (-0.24, 0.044) | 0.57 (0.42, 0.72) |
| Global | Both | Colon and rectum cancer | -0.031 (-0.17, 0.11) | -0.093 (-0.23, 0.042) | 0.82 (0.63, 1) |
| Global | Both | Esophageal cancer | -1.6 (-2, -1.3) | -1.7 (-2.1, -1.4) | -1.5 (-1.8, -1.1) |
| Global | Both | Eye cancer | 1.2 (1.1, 1.3) | 1.2 (1.1, 1.3) | 1.3 (1.1, 1.5) |
| Global | Both | Gallbladder and biliary tract cancer | -0.36 (-0.46, -0.26) | -0.38 (-0.48, -0.28) | 0.18 (0.035, 0.33) |
| Global | Both | Hodgkin lymphoma | -1.2 (-1.3, -1.1) | -1.1 (-1.2, -1.1) | -1.6 (-1.8, -1.4) |
| Global | Both | Kidney cancer | -0.53 (-0.66, -0.41) | -0.59 (-0.7, -0.49) | -0.11 (-0.33, 0.11) |
| Global | Both | Larynx cancer | -0.77 (-0.99, -0.55) | -0.81 (-1, -0.58) | -0.51 (-0.73, -0.3) |
| Global | Both | Leukemia | -1.1 (-1.2, -1.1) | -1.1 (-1.2, -1) | -0.77 (-0.85, -0.69) |
| Global | Both | Lip and oral cavity cancer | 0.88 (0.79, 0.98) | 0.86 (0.76, 0.96) | 1.5 (1.3, 1.6) |
| Global | Both | Liver cancer | -1 (-1.3, -0.81) | -1.1 (-1.4, -0.87) | -1 (-1.2, -0.85) |
| Global | Both | Malignant neoplasm of bone and articular cartilage | 0.03 (-0.011, 0.07) | 0.041 (-0.0025, 0.085) | 0.48 (0.38, 0.58) |
| Global | Both | Malignant skin melanoma | -1.5 (-1.7, -1.3) | -1.5 (-1.7, -1.4) | -2.1 (-2.4, -1.9) |
| Global | Both | Mesothelioma | -0.52 (-0.64, -0.41) | -0.57 (-0.69, -0.45) | -0.51 (-0.64, -0.38) |
| Global | Both | Multiple myeloma | 0.99 (0.84, 1.1) | 0.97 (0.83, 1.1) | 0.74 (0.59, 0.88) |
| Global | Both | Nasopharynx cancer | -0.39 (-0.46, -0.32) | -0.48 (-0.55, -0.41) | 1.6 (1.4, 1.8) |
| Global | Both | Neuroblastoma and other peripheral nervous cell tumors | 1.2 (1.1, 1.3) | 1.2 (1.1, 1.3) | 1.4 (1.2, 1.5) |
| Global | Both | Non-Hodgkin lymphoma | -0.16 (-0.22, -0.1) | -0.18 (-0.23, -0.12) | 0.45 (0.32, 0.59) |
| Global | Both | Non-melanoma skin cancer | -0.95 (-1, -0.86) | -1 (-1.1, -0.92) | 0.38 (-0.27, 1) |
| Global | Both | Other malignant neoplasms | -0.12 (-0.21, -0.033) | -0.096 (-0.18, -0.0087) | 0.72 (0.55, 0.9) |
| Global | Both | Other pharynx cancer | 1 (0.86, 1.2) | 1 (0.85, 1.2) | 1.5 (1.3, 1.6) |
| Global | Both | Ovarian cancer | 0.65 (0.58, 0.72) | 0.63 (0.56, 0.71) | 0.76 (0.69, 0.82) |
| Global | Both | Pancreatic cancer | -0.11 (-0.23, 0.012) | -0.17 (-0.29, -0.047) | -0.11 (-0.24, 0.022) |
| Global | Both | Prostate cancer | 0.21 (0.051, 0.37) | 0.21 (0.063, 0.36) | 0.28 (-0.036, 0.6) |
| Global | Both | Soft tissue and other extraosseous sarcomas | 0.17 (-0.06, 0.4) | 0.23 (-0.008, 0.46) | 0.63 (0.3, 0.96) |
| Global | Both | Stomach cancer | -2.4 (-2.6, -2.3) | -2.5 (-2.7, -2.4) | -2.1 (-2.2, -2) |
| Global | Both | Testicular cancer | 1.1 (0.79, 1.5) | 1.1 (0.8, 1.4) | 1.7 (1.3, 2.2) |
| Global | Both | Thyroid cancer | 0.2 (0.11, 0.28) | 0.13 (0.045, 0.22) | 0.77 (0.68, 0.86) |
| Global | Both | Tracheal, bronchus, and lung cancer | -1.9 (-2, -1.7) | -2 (-2.1, -1.8) | -1.8 (-2, -1.7) |
| Global | Both | Uterine cancer | -0.58 (-0.89, -0.28) | -0.66 (-0.97, -0.35) | 0.38 (0.1, 0.66) |
| Global | Female | Bladder cancer | -0.23 (-0.33, -0.14) | -0.27 (-0.36, -0.18) | 0.0098 (-0.1, 0.12) |
| Global | Female | Brain and central nervous system cancer | -0.25 (-0.31, -0.19) | -0.26 (-0.32, -0.2) | 0.22 (0.11, 0.33) |
| Global | Female | Breast cancer | 1.1 (1, 1.2) | 1 (0.96, 1.1) | 1.3 (1.2, 1.4) |
| Global | Female | Cervical cancer | -0.056 (-0.19, 0.079) | -0.054 (-0.2, 0.087) | 0.61 (0.46, 0.77) |
| Global | Female | Colon and rectum cancer | -0.18 (-0.3, -0.065) | -0.18 (-0.3, -0.052) | 0.48 (0.3, 0.65) |
| Global | Female | Esophageal cancer | -0.67 (-0.88, -0.45) | -0.65 (-0.86, -0.43) | -0.63 (-0.88, -0.38) |
| Global | Female | Eye cancer | 1.6 (1.5, 1.6) | 1.6 (1.5, 1.6) | 1.7 (1.5, 1.8) |
| Global | Female | Gallbladder and biliary tract cancer | -0.12 (-0.27, 0.028) | -0.1 (-0.25, 0.05) | 0.16 (0.025, 0.3) |
| Global | Female | Hodgkin lymphoma | -1.2 (-1.4, -1.1) | -1.2 (-1.3, -1) | -1.9 (-2.1, -1.7) |
| Global | Female | Kidney cancer | -0.51 (-0.63, -0.39) | -0.51 (-0.63, -0.39) | -0.072 (-0.27, 0.13) |
| Global | Female | Larynx cancer | 0.2 (0.054, 0.35) | 0.24 (0.091, 0.39) | 0.36 (0.23, 0.5) |
| Global | Female | Leukemia | -1.4 (-1.5, -1.3) | -1.3 (-1.4, -1.2) | -1.1 (-1.1, -0.97) |
| Global | Female | Lip and oral cavity cancer | 1.7 (1.5, 2) | 1.8 (1.6, 2) | 2.4 (2.2, 2.6) |
| Global | Female | Liver cancer | -0.48 (-0.59, -0.36) | -0.54 (-0.67, -0.4) | -0.48 (-0.51, -0.45) |
| Global | Female | Malignant neoplasm of bone and articular cartilage | -0.32 (-0.42, -0.22) | -0.27 (-0.35, -0.18) | 0.17 (0.098, 0.24) |
| Global | Female | Malignant skin melanoma | -1.3 (-1.4, -1.1) | -1.3 (-1.4, -1.1) | -2 (-2.3, -1.8) |
| Global | Female | Mesothelioma | 0.097 (0.013, 0.18) | 0.071 (-0.015, 0.16) | 0.19 (0.11, 0.26) |
| Global | Female | Multiple myeloma | 1 (0.96, 1.1) | 1.1 (0.98, 1.1) | 0.68 (0.59, 0.77) |
| Global | Female | Nasopharynx cancer | -0.68 (-0.84, -0.52) | -0.68 (-0.82, -0.53) | 0.51 (0.37, 0.65) |
| Global | Female | Neuroblastoma and other peripheral nervous cell tumors | 1 (0.9, 1.1) | 1.1 (0.95, 1.2) | 1.2 (0.99, 1.3) |
| Global | Female | Non-Hodgkin lymphoma | 0.37 (0.28, 0.45) | 0.4 (0.31, 0.48) | 0.78 (0.66, 0.91) |
| Global | Female | Non-melanoma skin cancer | -1.5 (-1.6, -1.3) | -1.5 (-1.7, -1.4) | 0.58 (-0.13, 1.3) |
| Global | Female | Other malignant neoplasms | 0.12 (0.037, 0.21) | 0.17 (0.082, 0.26) | 0.86 (0.69, 1) |
| Global | Female | Other pharynx cancer | 1.4 (1.3, 1.6) | 1.5 (1.3, 1.6) | 2.1 (1.9, 2.3) |
| Global | Female | Ovarian cancer | 0.69 (0.62, 0.76) | 0.68 (0.6, 0.76) | 0.8 (0.73, 0.87) |
| Global | Female | Pancreatic cancer | -0.086 (-0.22, 0.048) | -0.098 (-0.24, 0.041) | -0.071 (-0.22, 0.078) |
| Global | Female | Soft tissue and other extraosseous sarcomas | 0.21 (0.0098, 0.41) | 0.28 (0.072, 0.49) | 0.68 (0.38, 0.98) |
| Global | Female | Stomach cancer | -2 (-2.2, -1.9) | -2 (-2.2, -1.8) | -1.9 (-2, -1.7) |
| Global | Female | Thyroid cancer | 0.18 (0.036, 0.32) | 0.14 (0.0028, 0.29) | 0.68 (0.58, 0.78) |
| Global | Female | Tracheal, bronchus, and lung cancer | -1.2 (-1.4, -1) | -1.3 (-1.5, -1.1) | -1.1 (-1.4, -0.92) |
| Global | Female | Uterine cancer | -0.54 (-0.84, -0.23) | -0.61 (-0.93, -0.3) | 0.43 (0.14, 0.71) |
| Global | Male | Bladder cancer | -0.88 (-0.95, -0.81) | -1 (-1.1, -0.94) | -0.23 (-0.31, -0.16) |
| Global | Male | Brain and central nervous system cancer | -0.2 (-0.33, -0.064) | -0.18 (-0.31, -0.048) | 0.35 (0.17, 0.53) |
| Global | Male | Breast cancer | -0.15 (-0.22, -0.09) | -0.26 (-0.32, -0.19) | -0.032 (-0.23, 0.17) |
| Global | Male | Colon and rectum cancer | 0.061 (-0.093, 0.21) | -0.049 (-0.19, 0.097) | 1.1 (0.84, 1.3) |
| Global | Male | Esophageal cancer | -1.9 (-2.4, -1.5) | -2.1 (-2.5, -1.7) | -1.7 (-2.1, -1.3) |
| Global | Male | Eye cancer | 0.79 (0.69, 0.88) | 0.79 (0.71, 0.87) | 0.99 (0.77, 1.2) |
| Global | Male | Gallbladder and biliary tract cancer | -0.63 (-0.74, -0.52) | -0.69 (-0.79, -0.58) | 0.2 (-0.0055, 0.41) |
| Global | Male | Hodgkin lymphoma | -1.2 (-1.3, -1.1) | -1.2 (-1.2, -1.1) | -1.4 (-1.6, -1.2) |
| Global | Male | Kidney cancer | -0.57 (-0.72, -0.42) | -0.65 (-0.78, -0.52) | -0.15 (-0.4, 0.1) |
| Global | Male | Larynx cancer | -1 (-1.3, -0.78) | -1.1 (-1.3, -0.82) | -0.73 (-0.97, -0.5) |
| Global | Male | Leukemia | -0.97 (-1.1, -0.87) | -0.93 (-1, -0.83) | -0.57 (-0.68, -0.46) |
| Global | Male | Lip and oral cavity cancer | 0.45 (0.36, 0.54) | 0.41 (0.32, 0.51) | 0.94 (0.86, 1) |
| Global | Male | Liver cancer | -1.2 (-1.5, -0.96) | -1.3 (-1.6, -1) | -1.2 (-1.4, -0.98) |
| Global | Male | Malignant neoplasm of bone and articular cartilage | 0.22 (0.13, 0.32) | 0.21 (0.11, 0.31) | 0.65 (0.5, 0.8) |
| Global | Male | Malignant skin melanoma | -1.7 (-1.9, -1.5) | -1.7 (-1.9, -1.5) | -2.2 (-2.5, -1.9) |
| Global | Male | Mesothelioma | -0.89 (-1, -0.75) | -0.94 (-1.1, -0.79) | -0.92 (-1.1, -0.76) |
| Global | Male | Multiple myeloma | 0.93 (0.73, 1.1) | 0.89 (0.71, 1.1) | 0.76 (0.56, 0.97) |
| Global | Male | Nasopharynx cancer | -0.29 (-0.4, -0.18) | -0.42 (-0.52, -0.32) | 2 (1.7, 2.3) |
| Global | Male | Neuroblastoma and other peripheral nervous cell tumors | 1.3 (1.1, 1.5) | 1.3 (1.2, 1.5) | 1.5 (1.3, 1.7) |
| Global | Male | Non-Hodgkin lymphoma | -0.49 (-0.57, -0.42) | -0.53 (-0.6, -0.46) | 0.25 (0.077, 0.42) |
| Global | Male | Non-melanoma skin cancer | -0.56 (-0.74, -0.38) | -0.61 (-0.8, -0.43) | 0.16 (-0.44, 0.75) |
| Global | Male | Other malignant neoplasms | -0.34 (-0.46, -0.22) | -0.33 (-0.45, -0.22) | 0.59 (0.4, 0.78) |
| Global | Male | Other pharynx cancer | 0.89 (0.69, 1.1) | 0.87 (0.67, 1.1) | 1.2 (1, 1.4) |
| Global | Male | Pancreatic cancer | -0.15 (-0.27, -0.023) | -0.23 (-0.35, -0.11) | -0.15 (-0.28, -0.024) |
| Global | Male | Prostate cancer | 0.17 (0.01, 0.32) | 0.17 (0.022, 0.32) | 0.24 (-0.076, 0.56) |
| Global | Male | Soft tissue and other extraosseous sarcomas | 0.13 (-0.13, 0.39) | 0.18 (-0.085, 0.44) | 0.58 (0.22, 0.94) |
| Global | Male | Stomach cancer | -2.7 (-2.8, -2.5) | -2.8 (-3, -2.7) | -2.2 (-2.4, -2.1) |
| Global | Male | Testicular cancer | 1.1 (0.75, 1.4) | 1.1 (0.76, 1.4) | 1.7 (1.3, 2.1) |
| Global | Male | Thyroid cancer | 0.26 (0.17, 0.35) | 0.14 (0.065, 0.22) | 1 (0.85, 1.2) |
| Global | Male | Tracheal, bronchus, and lung cancer | -2.2 (-2.3, -2.1) | -2.3 (-2.5, -2.2) | -2.2 (-2.4, -2.1) |
| High SDI | Both | Bladder cancer | -1.2 (-1.5, -0.96) | -1.4 (-1.6, -1.1) | -0.78 (-1.1, -0.47) |
| High SDI | Both | Brain and central nervous system cancer | -0.47 (-0.84, -0.098) | -0.49 (-0.86, -0.11) | -0.041 (-0.42, 0.34) |
| High SDI | Both | Breast cancer | -1.1 (-1.3, -0.87) | -1.2 (-1.4, -1) | -0.85 (-1.1, -0.61) |
| High SDI | Both | Cervical cancer | -1.5 (-1.7, -1.3) | -1.6 (-1.8, -1.4) | -0.86 (-1.1, -0.59) |
| High SDI | Both | Colon and rectum cancer | -0.28 (-0.64, 0.081) | -0.32 (-0.67, 0.031) | 0.096 (-0.3, 0.49) |
| High SDI | Both | Esophageal cancer | -1.7 (-2, -1.5) | -1.9 (-2.1, -1.6) | -1.6 (-1.8, -1.4) |
| High SDI | Both | Eye cancer | 0.056 (-0.43, 0.55) | -0.11 (-0.58, 0.36) | 0.53 (-0.035, 1.1) |
| High SDI | Both | Gallbladder and biliary tract cancer | -0.84 (-1.1, -0.55) | -0.89 (-1.2, -0.6) | -0.096 (-0.5, 0.31) |
| High SDI | Both | Hodgkin lymphoma | -3.9 (-4.2, -3.6) | -3.9 (-4.2, -3.6) | -3.5 (-3.8, -3.2) |
| High SDI | Both | Kidney cancer | -1.6 (-1.9, -1.2) | -1.7 (-2, -1.4) | -1.1 (-1.4, -0.73) |
| High SDI | Both | Larynx cancer | -3.5 (-3.7, -3.3) | -3.6 (-3.8, -3.4) | -3.2 (-3.4, -3) |
| High SDI | Both | Leukemia | -1.8 (-2, -1.7) | -1.8 (-2, -1.6) | -1.7 (-1.8, -1.5) |
| High SDI | Both | Lip and oral cavity cancer | -1.5 (-1.7, -1.2) | -1.6 (-1.8, -1.4) | -0.9 (-1.2, -0.59) |
| High SDI | Both | Liver cancer | -1.7 (-2.1, -1.3) | -1.8 (-2.3, -1.4) | -1.9 (-2.3, -1.4) |
| High SDI | Both | Malignant neoplasm of bone and articular cartilage | 0.53 (0.23, 0.84) | 0.56 (0.25, 0.87) | 1 (0.65, 1.4) |
| High SDI | Both | Malignant skin melanoma | -3.1 (-3.4, -2.8) | -3.3 (-3.5, -3) | -2.7 (-3, -2.4) |
| High SDI | Both | Mesothelioma | -2.3 (-2.5, -2.1) | -2.4 (-2.6, -2.2) | -2.4 (-2.6, -2.2) |
| High SDI | Both | Multiple myeloma | -1.3 (-1.7, -0.96) | -1.4 (-1.7, -1) | -2 (-2.3, -1.7) |
| High SDI | Both | Nasopharynx cancer | -1.5 (-1.7, -1.3) | -1.5 (-1.7, -1.4) | -0.65 (-0.92, -0.38) |
| High SDI | Both | Neuroblastoma and other peripheral nervous cell tumors | -0.39 (-0.58, -0.2) | -0.37 (-0.57, -0.16) | -0.32 (-0.53, -0.11) |
| High SDI | Both | Non-Hodgkin lymphoma | -1.5 (-1.7, -1.4) | -1.6 (-1.7, -1.4) | -1 (-1.2, -0.78) |
| High SDI | Both | Non-melanoma skin cancer | -0.34 (-0.89, 0.21) | -0.48 (-1.1, 0.14) | -0.057 (-0.19, 0.079) |
| High SDI | Both | Other malignant neoplasms | -0.59 (-0.91, -0.27) | -0.67 (-0.99, -0.36) | 0.31 (-0.056, 0.67) |
| High SDI | Both | Other pharynx cancer | -0.64 (-1.1, -0.2) | -0.75 (-1.2, -0.32) | -0.12 (-0.62, 0.37) |
| High SDI | Both | Ovarian cancer | -1.7 (-1.9, -1.5) | -1.7 (-1.9, -1.6) | -1.6 (-1.8, -1.4) |
| High SDI | Both | Pancreatic cancer | -0.51 (-0.77, -0.25) | -0.57 (-0.83, -0.31) | -0.52 (-0.8, -0.24) |
| High SDI | Both | Prostate cancer | -1.1 (-1.6, -0.64) | -1.2 (-1.7, -0.74) | -1.1 (-1.8, -0.49) |
| High SDI | Both | Soft tissue and other extraosseous sarcomas | 0.84 (0.21, 1.5) | 0.77 (0.14, 1.4) | 1.1 (0.41, 1.7) |
| High SDI | Both | Stomach cancer | -2.5 (-2.7, -2.3) | -2.5 (-2.7, -2.3) | -2.4 (-2.6, -2.3) |
| High SDI | Both | Testicular cancer | 0.066 (-0.29, 0.42) | -0.15 (-0.51, 0.21) | 0.73 (0.36, 1.1) |
| High SDI | Both | Thyroid cancer | -1.3 (-1.5, -1.1) | -1.6 (-1.8, -1.4) | -0.88 (-1.1, -0.66) |
| High SDI | Both | Tracheal, bronchus, and lung cancer | -3.8 (-4.1, -3.6) | -4 (-4.2, -3.7) | -3.9 (-4.1, -3.7) |
| High SDI | Both | Uterine cancer | 1 (0.44, 1.6) | 0.92 (0.36, 1.5) | 1.5 (0.85, 2.1) |
| High SDI | Female | Bladder cancer | -0.69 (-0.97, -0.4) | -0.81 (-1.1, -0.52) | -0.3 (-0.63, 0.027) |
| High SDI | Female | Brain and central nervous system cancer | -0.66 (-1.1, -0.23) | -0.69 (-1.1, -0.26) | -0.25 (-0.67, 0.18) |
| High SDI | Female | Breast cancer | -1.1 (-1.3, -0.83) | -1.2 (-1.4, -0.97) | -0.81 (-1, -0.57) |
| High SDI | Female | Cervical cancer | -1.4 (-1.7, -1.2) | -1.5 (-1.7, -1.3) | -0.81 (-1.1, -0.54) |
| High SDI | Female | Colon and rectum cancer | -0.6 (-0.96, -0.24) | -0.65 (-1, -0.3) | -0.15 (-0.56, 0.25) |
| High SDI | Female | Esophageal cancer | -0.59 (-0.88, -0.3) | -0.67 (-0.96, -0.38) | -0.39 (-0.71, -0.073) |
| High SDI | Female | Eye cancer | -0.086 (-0.54, 0.37) | -0.25 (-0.68, 0.19) | 0.4 (-0.13, 0.93) |
| High SDI | Female | Gallbladder and biliary tract cancer | -0.88 (-1.1, -0.66) | -0.91 (-1.1, -0.68) | -0.27 (-0.57, 0.032) |
| High SDI | Female | Hodgkin lymphoma | -4.4 (-4.7, -4.1) | -4.4 (-4.7, -4.1) | -4.2 (-4.5, -3.9) |
| High SDI | Female | Kidney cancer | -1.6 (-1.9, -1.2) | -1.7 (-2.1, -1.3) | -1 (-1.5, -0.55) |
| High SDI | Female | Larynx cancer | -2 (-2.3, -1.7) | -2.1 (-2.4, -1.8) | -1.9 (-2.2, -1.6) |
| High SDI | Female | Leukemia | -2 (-2.2, -1.8) | -2 (-2.2, -1.8) | -1.8 (-2, -1.6) |
| High SDI | Female | Lip and oral cavity cancer | -0.54 (-0.89, -0.2) | -0.64 (-0.97, -0.31) | -0.099 (-0.52, 0.33) |
| High SDI | Female | Liver cancer | 0.026 (-0.24, 0.29) | -0.066 (-0.32, 0.19) | -0.43 (-0.54, -0.33) |
| High SDI | Female | Malignant neoplasm of bone and articular cartilage | 0.88 (0.53, 1.2) | 0.9 (0.55, 1.3) | 1.3 (0.93, 1.7) |
| High SDI | Female | Malignant skin melanoma | -3 (-3.2, -2.8) | -3.2 (-3.4, -3) | -2.6 (-2.8, -2.4) |
| High SDI | Female | Mesothelioma | -1.5 (-1.9, -1.1) | -1.6 (-2, -1.2) | -1.5 (-1.9, -1.1) |
| High SDI | Female | Multiple myeloma | -1.2 (-1.4, -0.97) | -1.2 (-1.4, -0.98) | -2.3 (-2.4, -2.1) |
| High SDI | Female | Nasopharynx cancer | -1.7 (-1.9, -1.6) | -1.8 (-2, -1.6) | -1 (-1.2, -0.88) |
| High SDI | Female | Neuroblastoma and other peripheral nervous cell tumors | -0.53 (-0.76, -0.3) | -0.5 (-0.74, -0.26) | -0.45 (-0.69, -0.21) |
| High SDI | Female | Non-Hodgkin lymphoma | -1.4 (-1.5, -1.3) | -1.5 (-1.6, -1.4) | -0.94 (-1.1, -0.8) |
| High SDI | Female | Non-melanoma skin cancer | -0.17 (-0.38, 0.035) | -0.28 (-0.53, -0.017) | 0.048 (-0.088, 0.18) |
| High SDI | Female | Other malignant neoplasms | -0.49 (-0.8, -0.18) | -0.61 (-0.92, -0.31) | 0.41 (0.065, 0.76) |
| High SDI | Female | Other pharynx cancer | 0.46 (-0.15, 1.1) | 0.4 (-0.22, 1) | 0.62 (-0.096, 1.3) |
| High SDI | Female | Ovarian cancer | -1.7 (-1.8, -1.5) | -1.7 (-1.9, -1.5) | -1.5 (-1.7, -1.4) |
| High SDI | Female | Pancreatic cancer | -0.36 (-0.63, -0.084) | -0.4 (-0.68, -0.12) | -0.35 (-0.65, -0.058) |
| High SDI | Female | Soft tissue and other extraosseous sarcomas | 0.85 (0.23, 1.5) | 0.75 (0.12, 1.4) | 1.1 (0.41, 1.7) |
| High SDI | Female | Stomach cancer | -2.5 (-2.7, -2.2) | -2.4 (-2.7, -2.2) | -2.4 (-2.7, -2.2) |
| High SDI | Female | Thyroid cancer | -1.6 (-1.8, -1.4) | -1.9 (-2.1, -1.7) | -1.2 (-1.4, -1) |
| High SDI | Female | Tracheal, bronchus, and lung cancer | -3.8 (-4.2, -3.5) | -3.9 (-4.3, -3.6) | -3.8 (-4.1, -3.5) |
| High SDI | Female | Uterine cancer | 1.1 (0.48, 1.6) | 0.96 (0.4, 1.5) | 1.5 (0.89, 2.2) |
| High SDI | Male | Bladder cancer | -1.5 (-1.8, -1.2) | -1.7 (-1.9, -1.4) | -1 (-1.3, -0.7) |
| High SDI | Male | Brain and central nervous system cancer | -0.37 (-0.71, -0.017) | -0.38 (-0.73, -0.027) | 0.11 (-0.25, 0.46) |
| High SDI | Male | Breast cancer | -0.61 (-1.2, -0.061) | -0.7 (-1.2, -0.16) | -0.58 (-1.2, 0.078) |
| High SDI | Male | Colon and rectum cancer | -0.053 (-0.42, 0.32) | -0.092 (-0.46, 0.28) | 0.28 (-0.11, 0.68) |
| High SDI | Male | Esophageal cancer | -2 (-2.2, -1.7) | -2.1 (-2.4, -1.8) | -1.8 (-2.1, -1.6) |
| High SDI | Male | Eye cancer | 0.18 (-0.35, 0.7) | 0.0028 (-0.51, 0.51) | 0.63 (0.042, 1.2) |
| High SDI | Male | Gallbladder and biliary tract cancer | -0.81 (-1.2, -0.39) | -0.86 (-1.3, -0.45) | 0.049 (-0.49, 0.59) |
| High SDI | Male | Hodgkin lymphoma | -3.6 (-3.9, -3.3) | -3.6 (-3.9, -3.3) | -3 (-3.3, -2.6) |
| High SDI | Male | Kidney cancer | -1.6 (-1.9, -1.3) | -1.7 (-2, -1.4) | -1.2 (-1.5, -0.8) |
| High SDI | Male | Larynx cancer | -3.8 (-4, -3.6) | -3.9 (-4.2, -3.7) | -3.5 (-3.8, -3.3) |
| High SDI | Male | Leukemia | -1.7 (-1.9, -1.6) | -1.7 (-1.9, -1.5) | -1.6 (-1.7, -1.5) |
| High SDI | Male | Lip and oral cavity cancer | -1.8 (-2, -1.6) | -1.9 (-2.1, -1.7) | -1.3 (-1.6, -1) |
| High SDI | Male | Liver cancer | -2.3 (-2.9, -1.8) | -2.4 (-3, -1.9) | -2.3 (-2.9, -1.8) |
| High SDI | Male | Malignant neoplasm of bone and articular cartilage | 0.32 (0.025, 0.61) | 0.35 (0.048, 0.66) | 0.8 (0.46, 1.1) |
| High SDI | Male | Malignant skin melanoma | -3.2 (-3.5, -2.8) | -3.3 (-3.6, -3) | -2.9 (-3.2, -2.5) |
| High SDI | Male | Mesothelioma | -2.7 (-2.9, -2.5) | -2.8 (-3, -2.5) | -2.8 (-3.1, -2.6) |
| High SDI | Male | Multiple myeloma | -1.4 (-1.8, -0.95) | -1.5 (-1.9, -1) | -1.8 (-2.2, -1.4) |
| High SDI | Male | Nasopharynx cancer | -1.4 (-1.6, -1.2) | -1.5 (-1.7, -1.3) | -0.53 (-0.87, -0.2) |
| High SDI | Male | Neuroblastoma and other peripheral nervous cell tumors | -0.28 (-0.49, -0.068) | -0.26 (-0.48, -0.04) | -0.21 (-0.44, 0.024) |
| High SDI | Male | Non-Hodgkin lymphoma | -1.6 (-1.8, -1.4) | -1.6 (-1.8, -1.5) | -1.1 (-1.4, -0.78) |
| High SDI | Male | Non-melanoma skin cancer | -0.44 (-1.2, 0.32) | -0.6 (-1.4, 0.23) | -0.17 (-0.31, -0.037) |
| High SDI | Male | Other malignant neoplasms | -0.68 (-1, -0.33) | -0.73 (-1.1, -0.38) | 0.19 (-0.24, 0.62) |
| High SDI | Male | Other pharynx cancer | -0.9 (-1.3, -0.46) | -1 (-1.4, -0.58) | -0.34 (-0.84, 0.15) |
| High SDI | Male | Pancreatic cancer | -0.61 (-0.89, -0.34) | -0.69 (-0.96, -0.41) | -0.63 (-0.92, -0.34) |
| High SDI | Male | Prostate cancer | -1.2 (-1.7, -0.68) | -1.3 (-1.7, -0.78) | -1.2 (-1.8, -0.53) |
| High SDI | Male | Soft tissue and other extraosseous sarcomas | 0.83 (0.19, 1.5) | 0.77 (0.13, 1.4) | 1.1 (0.39, 1.8) |
| High SDI | Male | Stomach cancer | -2.5 (-2.7, -2.3) | -2.6 (-2.8, -2.3) | -2.4 (-2.6, -2.2) |
| High SDI | Male | Testicular cancer | 0.023 (-0.33, 0.38) | -0.19 (-0.55, 0.16) | 0.69 (0.32, 1.1) |
| High SDI | Male | Thyroid cancer | -0.95 (-1.3, -0.63) | -1.3 (-1.6, -1) | -0.2 (-0.57, 0.17) |
| High SDI | Male | Tracheal, bronchus, and lung cancer | -3.9 (-4.1, -3.7) | -4 (-4.2, -3.8) | -4 (-4.2, -3.8) |
| High-middle SDI | Both | Bladder cancer | -0.89 (-1, -0.77) | -1.1 (-1.2, -0.94) | 0.42 (0.28, 0.56) |
| High-middle SDI | Both | Brain and central nervous system cancer | 0.17 (-0.045, 0.38) | 0.23 (0.015, 0.44) | 1.2 (0.92, 1.4) |
| High-middle SDI | Both | Breast cancer | 0.28 (0.12, 0.43) | 0.16 (0.0072, 0.3) | 1.6 (1.4, 1.8) |
| High-middle SDI | Both | Cervical cancer | -0.49 (-0.78, -0.21) | -0.51 (-0.79, -0.22) | 0.64 (0.27, 1) |
| High-middle SDI | Both | Colon and rectum cancer | 0.7 (0.52, 0.89) | 0.58 (0.41, 0.75) | 2.4 (2.1, 2.7) |
| High-middle SDI | Both | Esophageal cancer | -1.5 (-2.1, -0.99) | -1.7 (-2.2, -1.1) | -0.85 (-1.4, -0.33) |
| High-middle SDI | Both | Eye cancer | -0.92 (-1.2, -0.65) | -0.89 (-1.1, -0.64) | -0.21 (-0.6, 0.19) |
| High-middle SDI | Both | Gallbladder and biliary tract cancer | -0.7 (-0.84, -0.57) | -0.77 (-0.9, -0.64) | 0.87 (0.66, 1.1) |
| High-middle SDI | Both | Hodgkin lymphoma | -3.2 (-3.4, -2.9) | -3 (-3.3, -2.8) | -1.9 (-2.3, -1.5) |
| High-middle SDI | Both | Kidney cancer | -0.3 (-0.45, -0.14) | -0.38 (-0.52, -0.24) | 1 (0.73, 1.3) |
| High-middle SDI | Both | Larynx cancer | -1.5 (-1.8, -1.3) | -1.6 (-1.8, -1.3) | -0.39 (-0.58, -0.19) |
| High-middle SDI | Both | Leukemia | -1.2 (-1.3, -1) | -1.1 (-1.3, -0.95) | -0.057 (-0.23, 0.12) |
| High-middle SDI | Both | Lip and oral cavity cancer | 0.62 (0.38, 0.86) | 0.61 (0.36, 0.86) | 2 (1.6, 2.3) |
| High-middle SDI | Both | Liver cancer | -0.44 (-0.66, -0.23) | -0.54 (-0.8, -0.27) | -0.36 (-0.48, -0.24) |
| High-middle SDI | Both | Malignant neoplasm of bone and articular cartilage | -1.2 (-1.4, -1) | -1.2 (-1.3, -0.98) | -0.23 (-0.45, -0.017) |
| High-middle SDI | Both | Malignant skin melanoma | -0.94 (-1.3, -0.54) | -0.92 (-1.3, -0.53) | 0.071 (-0.53, 0.68) |
| High-middle SDI | Both | Mesothelioma | -0.84 (-1, -0.65) | -0.82 (-1, -0.63) | -0.66 (-0.85, -0.47) |
| High-middle SDI | Both | Multiple myeloma | 1.3 (1, 1.6) | 1.3 (1, 1.5) | 1.6 (1.3, 1.9) |
| High-middle SDI | Both | Nasopharynx cancer | 0.88 (0.64, 1.1) | 0.62 (0.41, 0.82) | 4.5 (4, 5.1) |
| High-middle SDI | Both | Neuroblastoma and other peripheral nervous cell tumors | 1.5 (1.3, 1.8) | 1.7 (1.4, 2) | 2.1 (1.8, 2.4) |
| High-middle SDI | Both | Non-Hodgkin lymphoma | -0.36 (-0.57, -0.15) | -0.41 (-0.6, -0.21) | 1.4 (1.1, 1.8) |
| High-middle SDI | Both | Non-melanoma skin cancer | -1.4 (-1.5, -1.2) | -1.4 (-1.6, -1.3) | 6.1 (2.8, 9.5) |
| High-middle SDI | Both | Other malignant neoplasms | -1.1 (-1.5, -0.77) | -1.1 (-1.4, -0.77) | 1.5 (1.1, 1.9) |
| High-middle SDI | Both | Other pharynx cancer | 1 (0.74, 1.3) | 0.99 (0.71, 1.3) | 2.4 (2, 2.8) |
| High-middle SDI | Both | Ovarian cancer | -0.31 (-0.46, -0.17) | -0.25 (-0.4, -0.09) | -0.13 (-0.28, 0.019) |
| High-middle SDI | Both | Pancreatic cancer | 0.69 (0.51, 0.88) | 0.64 (0.46, 0.82) | 0.78 (0.59, 0.96) |
| High-middle SDI | Both | Prostate cancer | -0.0088 (-0.37, 0.36) | -0.17 (-0.54, 0.19) | 2 (1.8, 2.3) |
| High-middle SDI | Both | Soft tissue and other extraosseous sarcomas | 0.66 (0.075, 1.2) | 0.75 (0.18, 1.3) | 1.3 (0.61, 1.9) |
| High-middle SDI | Both | Stomach cancer | -2 (-2.2, -1.9) | -2.2 (-2.3, -2) | -1.1 (-1.3, -1) |
| High-middle SDI | Both | Testicular cancer | 0.62 (0.06, 1.2) | 0.44 (-0.11, 1) | 2.8 (2.2, 3.4) |
| High-middle SDI | Both | Thyroid cancer | -1.4 (-1.6, -1.1) | -1.6 (-1.8, -1.4) | -0.11 (-0.42, 0.19) |
| High-middle SDI | Both | Tracheal, bronchus, and lung cancer | -1.2 (-1.4, -1) | -1.3 (-1.5, -1.1) | -0.86 (-1, -0.68) |
| High-middle SDI | Both | Uterine cancer | -0.94 (-1.5, -0.41) | -1.1 (-1.6, -0.52) | 0.69 (0.26, 1.1) |
| High-middle SDI | Female | Bladder cancer | -0.77 (-0.99, -0.56) | -0.82 (-1, -0.61) | 0.42 (0.2, 0.64) |
| High-middle SDI | Female | Brain and central nervous system cancer | -0.12 (-0.28, 0.047) | -0.039 (-0.2, 0.12) | 1 (0.82, 1.2) |
| High-middle SDI | Female | Breast cancer | 0.47 (0.31, 0.62) | 0.35 (0.19, 0.5) | 1.8 (1.6, 2) |
| High-middle SDI | Female | Cervical cancer | -0.32 (-0.6, -0.036) | -0.33 (-0.62, -0.048) | 0.81 (0.44, 1.2) |
| High-middle SDI | Female | Colon and rectum cancer | -0.081 (-0.27, 0.11) | -0.1 (-0.29, 0.09) | 1.5 (1.3, 1.8) |
| High-middle SDI | Female | Esophageal cancer | -2.1 (-2.8, -1.4) | -2.1 (-2.8, -1.4) | -1.2 (-1.9, -0.49) |
| High-middle SDI | Female | Eye cancer | -0.78 (-1.1, -0.5) | -0.69 (-0.95, -0.43) | -0.12 (-0.51, 0.28) |
| High-middle SDI | Female | Gallbladder and biliary tract cancer | -1.1 (-1.4, -0.86) | -1.1 (-1.4, -0.84) | 0.11 (-0.15, 0.37) |
| High-middle SDI | Female | Hodgkin lymphoma | -3.5 (-3.7, -3.2) | -3.3 (-3.5, -3) | -2.2 (-2.6, -1.9) |
| High-middle SDI | Female | Kidney cancer | -0.99 (-1.1, -0.87) | -1 (-1.2, -0.91) | 0.55 (0.36, 0.74) |
| High-middle SDI | Female | Larynx cancer | -0.65 (-0.89, -0.41) | -0.58 (-0.83, -0.33) | 0.3 (0.093, 0.51) |
| High-middle SDI | Female | Leukemia | -1.9 (-2.1, -1.7) | -1.8 (-1.9, -1.6) | -0.74 (-0.92, -0.55) |
| High-middle SDI | Female | Lip and oral cavity cancer | 1.4 (1, 1.8) | 1.5 (1.1, 1.9) | 2.8 (2.3, 3.2) |
| High-middle SDI | Female | Liver cancer | -1.4 (-1.5, -1.2) | -1.4 (-1.6, -1.2) | -1.1 (-1.2, -0.95) |
| High-middle SDI | Female | Malignant neoplasm of bone and articular cartilage | -1.7 (-1.9, -1.5) | -1.6 (-1.8, -1.4) | -0.69 (-0.92, -0.47) |
| High-middle SDI | Female | Malignant skin melanoma | -1 (-1.4, -0.63) | -0.98 (-1.3, -0.63) | 0.026 (-0.59, 0.64) |
| High-middle SDI | Female | Mesothelioma | -0.72 (-0.93, -0.51) | -0.64 (-0.85, -0.42) | -0.44 (-0.65, -0.24) |
| High-middle SDI | Female | Multiple myeloma | 1.1 (0.9, 1.3) | 1.2 (0.96, 1.4) | 1.5 (1.2, 1.7) |
| High-middle SDI | Female | Nasopharynx cancer | -0.43 (-0.7, -0.16) | -0.54 (-0.81, -0.26) | 2.2 (2, 2.5) |
| High-middle SDI | Female | Neuroblastoma and other peripheral nervous cell tumors | 1.1 (0.84, 1.4) | 1.3 (1, 1.6) | 1.5 (1.3, 1.8) |
| High-middle SDI | Female | Non-Hodgkin lymphoma | -0.34 (-0.54, -0.15) | -0.29 (-0.48, -0.11) | 1.4 (1.1, 1.7) |
| High-middle SDI | Female | Non-melanoma skin cancer | -2 (-2.2, -1.9) | -2 (-2.1, -1.9) | 6.7 (3.3, 10) |
| High-middle SDI | Female | Other malignant neoplasms | -1.1 (-1.4, -0.76) | -1 (-1.3, -0.73) | 1.6 (1.2, 2) |
| High-middle SDI | Female | Other pharynx cancer | 1.9 (1.5, 2.4) | 2 (1.6, 2.5) | 3.5 (3, 4.1) |
| High-middle SDI | Female | Ovarian cancer | -0.14 (-0.3, 0.011) | -0.073 (-0.23, 0.088) | 0.044 (-0.11, 0.19) |
| High-middle SDI | Female | Pancreatic cancer | 0.35 (0.14, 0.56) | 0.38 (0.17, 0.59) | 0.5 (0.28, 0.72) |
| High-middle SDI | Female | Soft tissue and other extraosseous sarcomas | 0.64 (0.034, 1.3) | 0.77 (0.18, 1.4) | 1.3 (0.6, 2) |
| High-middle SDI | Female | Stomach cancer | -2.3 (-2.6, -2.1) | -2.3 (-2.6, -2.1) | -1.5 (-1.8, -1.3) |
| High-middle SDI | Female | Thyroid cancer | -2 (-2.3, -1.7) | -2.3 (-2.5, -2.1) | -0.64 (-0.97, -0.32) |
| High-middle SDI | Female | Tracheal, bronchus, and lung cancer | -0.46 (-0.71, -0.2) | -0.47 (-0.72, -0.21) | 0.16 (-0.1, 0.42) |
| High-middle SDI | Female | Uterine cancer | -0.77 (-1.3, -0.23) | -0.9 (-1.5, -0.34) | 0.86 (0.43, 1.3) |
| High-middle SDI | Male | Bladder cancer | -1 (-1.2, -0.92) | -1.3 (-1.4, -1.1) | 0.3 (0.16, 0.43) |
| High-middle SDI | Male | Brain and central nervous system cancer | 0.3 (0.051, 0.56) | 0.35 (0.099, 0.6) | 1.3 (0.98, 1.6) |
| High-middle SDI | Male | Breast cancer | -1.1 (-1.3, -0.98) | -1.4 (-1.6, -1.3) | 0.32 (0.023, 0.62) |
| High-middle SDI | Male | Colon and rectum cancer | 1.1 (0.84, 1.3) | 0.89 (0.68, 1.1) | 2.9 (2.5, 3.2) |
| High-middle SDI | Male | Esophageal cancer | -1.6 (-2.1, -1.1) | -1.7 (-2.2, -1.2) | -0.95 (-1.4, -0.45) |
| High-middle SDI | Male | Eye cancer | -1.1 (-1.4, -0.77) | -1.1 (-1.4, -0.82) | -0.29 (-0.71, 0.14) |
| High-middle SDI | Male | Gallbladder and biliary tract cancer | -0.4 (-0.61, -0.19) | -0.53 (-0.73, -0.34) | 1.4 (1, 1.7) |
| High-middle SDI | Male | Hodgkin lymphoma | -3 (-3.4, -2.7) | -2.9 (-3.3, -2.6) | -1.7 (-2.1, -1.2) |
| High-middle SDI | Male | Kidney cancer | -0.17 (-0.35, 0.018) | -0.28 (-0.44, -0.12) | 1.1 (0.8, 1.5) |
| High-middle SDI | Male | Larynx cancer | -1.8 (-2, -1.5) | -1.9 (-2.1, -1.6) | -0.62 (-0.82, -0.42) |
| High-middle SDI | Male | Leukemia | -0.72 (-0.92, -0.52) | -0.68 (-0.87, -0.49) | 0.37 (0.15, 0.58) |
| High-middle SDI | Male | Lip and oral cavity cancer | 0.28 (0.061, 0.49) | 0.25 (0.03, 0.47) | 1.5 (1.3, 1.8) |
| High-middle SDI | Male | Liver cancer | -0.39 (-0.63, -0.16) | -0.51 (-0.79, -0.22) | -0.36 (-0.48, -0.24) |
| High-middle SDI | Male | Malignant neoplasm of bone and articular cartilage | -0.99 (-1.2, -0.78) | -0.98 (-1.2, -0.79) | -0.041 (-0.28, 0.2) |
| High-middle SDI | Male | Malignant skin melanoma | -0.91 (-1.3, -0.46) | -0.89 (-1.3, -0.45) | 0.21 (-0.42, 0.83) |
| High-middle SDI | Male | Mesothelioma | -0.97 (-1.2, -0.8) | -0.99 (-1.2, -0.8) | -0.84 (-1, -0.65) |
| High-middle SDI | Male | Multiple myeloma | 1.4 (1, 1.7) | 1.3 (0.96, 1.6) | 1.6 (1.2, 2) |
| High-middle SDI | Male | Nasopharynx cancer | 1.2 (0.84, 1.5) | 0.85 (0.56, 1.1) | 5.3 (4.5, 6) |
| High-middle SDI | Male | Neuroblastoma and other peripheral nervous cell tumors | 1.9 (1.5, 2.2) | 2 (1.6, 2.3) | 2.5 (2, 2.9) |
| High-middle SDI | Male | Non-Hodgkin lymphoma | -0.45 (-0.71, -0.18) | -0.54 (-0.78, -0.3) | 1.4 (0.96, 1.8) |
| High-middle SDI | Male | Non-melanoma skin cancer | -0.91 (-1.2, -0.61) | -0.97 (-1.3, -0.66) | 5.4 (2.2, 8.6) |
| High-middle SDI | Male | Other malignant neoplasms | -1.2 (-1.6, -0.81) | -1.2 (-1.5, -0.83) | 1.4 (0.96, 1.8) |
| High-middle SDI | Male | Other pharynx cancer | 0.73 (0.47, 0.98) | 0.69 (0.43, 0.96) | 2 (1.7, 2.4) |
| High-middle SDI | Male | Pancreatic cancer | 0.73 (0.55, 0.92) | 0.64 (0.47, 0.81) | 0.79 (0.61, 0.97) |
| High-middle SDI | Male | Prostate cancer | -0.17 (-0.53, 0.18) | -0.34 (-0.69, 0.016) | 1.9 (1.6, 2.2) |
| High-middle SDI | Male | Soft tissue and other extraosseous sarcomas | 0.62 (0.059, 1.2) | 0.69 (0.14, 1.2) | 1.2 (0.58, 1.8) |
| High-middle SDI | Male | Stomach cancer | -2 (-2.1, -1.9) | -2.2 (-2.3, -2.1) | -1.1 (-1.2, -0.95) |
| High-middle SDI | Male | Testicular cancer | 0.45 (-0.11, 1) | 0.28 (-0.27, 0.84) | 2.6 (2, 3.3) |
| High-middle SDI | Male | Thyroid cancer | -0.64 (-0.86, -0.42) | -0.94 (-1.1, -0.76) | 1.1 (0.79, 1.4) |
| High-middle SDI | Male | Tracheal, bronchus, and lung cancer | -1.6 (-1.8, -1.5) | -1.7 (-1.9, -1.6) | -1.5 (-1.6, -1.3) |
| Low SDI | Both | Bladder cancer | 0.45 (0.41, 0.5) | 0.48 (0.43, 0.53) | 1.3 (1.3, 1.4) |
| Low SDI | Both | Brain and central nervous system cancer | 1.2 (1.1, 1.3) | 1.3 (1.2, 1.4) | 1.7 (1.6, 1.8) |
| Low SDI | Both | Breast cancer | 1.8 (1.7, 1.9) | 1.9 (1.8, 1.9) | 2.9 (2.8, 3) |
| Low SDI | Both | Cervical cancer | -0.81 (-0.94, -0.69) | -0.77 (-0.89, -0.64) | 0.061 (-0.087, 0.21) |
| Low SDI | Both | Colon and rectum cancer | 0.38 (0.28, 0.48) | 0.44 (0.34, 0.53) | 1 (0.91, 1.1) |
| Low SDI | Both | Esophageal cancer | -0.046 (-0.11, 0.016) | 0.011 (-0.051, 0.074) | 0.067 (0.0021, 0.13) |
| Low SDI | Both | Eye cancer | 0.46 (0.41, 0.51) | 0.45 (0.4, 0.5) | 1.3 (1.2, 1.4) |
| Low SDI | Both | Gallbladder and biliary tract cancer | 0.09 (-0.17, 0.35) | 0.18 (-0.086, 0.45) | 0.34 (0.07, 0.61) |
| Low SDI | Both | Hodgkin lymphoma | -1.3 (-1.4, -1.2) | -1.3 (-1.3, -1.2) | -0.29 (-0.4, -0.18) |
| Low SDI | Both | Kidney cancer | 1.4 (1.3, 1.4) | 1.4 (1.4, 1.5) | 2.4 (2.3, 2.4) |
| Low SDI | Both | Larynx cancer | -0.45 (-0.61, -0.29) | -0.42 (-0.58, -0.26) | 0.097 (-0.088, 0.28) |
| Low SDI | Both | Leukemia | -0.47 (-0.6, -0.33) | -0.38 (-0.51, -0.25) | -0.22 (-0.36, -0.086) |
| Low SDI | Both | Lip and oral cavity cancer | 0.32 (0.2, 0.43) | 0.34 (0.23, 0.44) | 1.3 (1.2, 1.5) |
| Low SDI | Both | Liver cancer | -1 (-1.2, -0.85) | -1 (-1.2, -0.85) | -0.98 (-1.1, -0.84) |
| Low SDI | Both | Malignant neoplasm of bone and articular cartilage | 0.051 (-0.077, 0.18) | 0.093 (-0.03, 0.22) | 0.49 (0.35, 0.64) |
| Low SDI | Both | Malignant skin melanoma | 0.7 (0.65, 0.74) | 0.7 (0.66, 0.74) | 1.9 (1.8, 2) |
| Low SDI | Both | Mesothelioma | 1.2 (1.1, 1.3) | 1.2 (1.1, 1.3) | 1.3 (1.3, 1.4) |
| Low SDI | Both | Multiple myeloma | 2 (2, 2.1) | 2.1 (2, 2.1) | 2.4 (2.3, 2.4) |
| Low SDI | Both | Nasopharynx cancer | -0.29 (-0.42, -0.15) | -0.21 (-0.35, -0.082) | 0.15 (0.0042, 0.29) |
| Low SDI | Both | Neuroblastoma and other peripheral nervous cell tumors | 3.9 (3.7, 4) | 3.9 (3.8, 4.1) | 4.6 (4.3, 4.9) |
| Low SDI | Both | Non-Hodgkin lymphoma | 0.37 (0.29, 0.44) | 0.42 (0.35, 0.49) | 1.2 (1.1, 1.3) |
| Low SDI | Both | Non-melanoma skin cancer | 0.82 (0.67, 0.96) | 0.85 (0.71, 1) | 0.28 (0.24, 0.32) |
| Low SDI | Both | Other malignant neoplasms | -0.0017 (-0.13, 0.13) | 0.075 (-0.048, 0.2) | 0.55 (0.4, 0.69) |
| Low SDI | Both | Other pharynx cancer | 0.29 (0.061, 0.53) | 0.32 (0.09, 0.55) | 1.1 (0.8, 1.3) |
| Low SDI | Both | Ovarian cancer | 2.4 (2.3, 2.5) | 2.4 (2.4, 2.5) | 2.7 (2.6, 2.9) |
| Low SDI | Both | Pancreatic cancer | 1.5 (1.4, 1.6) | 1.5 (1.5, 1.6) | 1.6 (1.5, 1.6) |
| Low SDI | Both | Prostate cancer | 1.1 (0.98, 1.2) | 1 (0.95, 1.1) | 2.3 (2.3, 2.3) |
| Low SDI | Both | Soft tissue and other extraosseous sarcomas | -0.67 (-0.78, -0.57) | -0.61 (-0.7, -0.51) | 0.034 (-0.079, 0.15) |
| Low SDI | Both | Stomach cancer | -1.2 (-1.4, -1.1) | -1.1 (-1.3, -1) | -1 (-1.2, -0.89) |
| Low SDI | Both | Testicular cancer | 0.99 (0.89, 1.1) | 0.96 (0.86, 1.1) | 3.1 (3, 3.2) |
| Low SDI | Both | Thyroid cancer | 0.2 (0.048, 0.35) | 0.19 (0.042, 0.33) | 2.4 (2.1, 2.6) |
| Low SDI | Both | Tracheal, bronchus, and lung cancer | 0.59 (0.5, 0.68) | 0.64 (0.55, 0.73) | 0.68 (0.59, 0.77) |
| Low SDI | Both | Uterine cancer | 0.89 (0.83, 0.95) | 0.92 (0.86, 0.97) | 2.2 (2.1, 2.3) |
| Low SDI | Female | Bladder cancer | 0.29 (0.23, 0.35) | 0.34 (0.28, 0.4) | 1.1 (1, 1.2) |
| Low SDI | Female | Brain and central nervous system cancer | 1.5 (1.4, 1.7) | 1.5 (1.4, 1.7) | 2 (1.8, 2.1) |
| Low SDI | Female | Breast cancer | 1.8 (1.7, 1.9) | 1.9 (1.8, 1.9) | 2.9 (2.8, 3) |
| Low SDI | Female | Cervical cancer | -0.85 (-0.97, -0.72) | -0.8 (-0.93, -0.67) | 0.027 (-0.12, 0.17) |
| Low SDI | Female | Colon and rectum cancer | 0.27 (0.15, 0.38) | 0.34 (0.22, 0.46) | 0.97 (0.84, 1.1) |
| Low SDI | Female | Esophageal cancer | -0.3 (-0.38, -0.21) | -0.21 (-0.3, -0.13) | -0.13 (-0.22, -0.042) |
| Low SDI | Female | Eye cancer | 0.81 (0.76, 0.85) | 0.82 (0.77, 0.86) | 1.7 (1.6, 1.8) |
| Low SDI | Female | Gallbladder and biliary tract cancer | 0.019 (-0.27, 0.31) | 0.13 (-0.19, 0.44) | 0.28 (-0.041, 0.59) |
| Low SDI | Female | Hodgkin lymphoma | -1.2 (-1.4, -1.1) | -1.2 (-1.4, -1.1) | -0.12 (-0.36, 0.12) |
| Low SDI | Female | Kidney cancer | 1.4 (1.3, 1.4) | 1.5 (1.4, 1.5) | 2.5 (2.5, 2.6) |
| Low SDI | Female | Larynx cancer | 0.093 (-0.09, 0.28) | 0.17 (-0.017, 0.36) | 0.68 (0.46, 0.91) |
| Low SDI | Female | Leukemia | -0.46 (-0.64, -0.29) | -0.38 (-0.54, -0.22) | -0.23 (-0.4, -0.067) |
| Low SDI | Female | Lip and oral cavity cancer | 0.82 (0.63, 1) | 0.86 (0.69, 1) | 1.9 (1.7, 2.1) |
| Low SDI | Female | Liver cancer | -0.16 (-0.29, -0.032) | -0.11 (-0.24, 0.021) | -0.037 (-0.14, 0.067) |
| Low SDI | Female | Malignant neoplasm of bone and articular cartilage | -0.25 (-0.46, -0.045) | -0.19 (-0.39, 0.0092) | 0.18 (-0.038, 0.39) |
| Low SDI | Female | Malignant skin melanoma | 0.94 (0.88, 1) | 0.96 (0.9, 1) | 2.4 (2.2, 2.5) |
| Low SDI | Female | Mesothelioma | 1.6 (1.5, 1.7) | 1.7 (1.6, 1.7) | 1.8 (1.7, 1.9) |
| Low SDI | Female | Multiple myeloma | 2.5 (2.4, 2.6) | 2.5 (2.5, 2.6) | 2.9 (2.8, 3) |
| Low SDI | Female | Nasopharynx cancer | -0.57 (-0.8, -0.35) | -0.47 (-0.69, -0.25) | -0.034 (-0.27, 0.21) |
| Low SDI | Female | Neuroblastoma and other peripheral nervous cell tumors | 3.6 (3.4, 3.8) | 3.7 (3.5, 3.9) | 4.4 (4.1, 4.7) |
| Low SDI | Female | Non-Hodgkin lymphoma | 1.2 (1.1, 1.3) | 1.3 (1.2, 1.4) | 2.1 (2, 2.2) |
| Low SDI | Female | Non-melanoma skin cancer | 0.51 (0.24, 0.78) | 0.58 (0.32, 0.85) | 0.34 (0.27, 0.42) |
| Low SDI | Female | Other malignant neoplasms | 0.037 (-0.12, 0.19) | 0.12 (-0.022, 0.27) | 0.65 (0.48, 0.82) |
| Low SDI | Female | Other pharynx cancer | 0.45 (0.25, 0.64) | 0.48 (0.28, 0.68) | 1.4 (1.1, 1.6) |
| Low SDI | Female | Ovarian cancer | 2.4 (2.3, 2.5) | 2.4 (2.3, 2.5) | 2.7 (2.6, 2.8) |
| Low SDI | Female | Pancreatic cancer | 1.6 (1.6, 1.7) | 1.7 (1.6, 1.8) | 1.8 (1.7, 1.9) |
| Low SDI | Female | Soft tissue and other extraosseous sarcomas | -0.66 (-0.8, -0.52) | -0.58 (-0.72, -0.45) | 0.062 (-0.093, 0.22) |
| Low SDI | Female | Stomach cancer | -1.2 (-1.3, -1) | -1.1 (-1.2, -0.93) | -0.97 (-1.1, -0.83) |
| Low SDI | Female | Thyroid cancer | -0.03 (-0.23, 0.17) | -0.067 (-0.25, 0.12) | 2.2 (2, 2.5) |
| Low SDI | Female | Tracheal, bronchus, and lung cancer | 1.7 (1.5, 1.8) | 1.7 (1.6, 1.8) | 1.8 (1.7, 1.9) |
| Low SDI | Female | Uterine cancer | 0.85 (0.79, 0.91) | 0.88 (0.83, 0.94) | 2.2 (2.1, 2.3) |
| Low SDI | Male | Bladder cancer | 0.58 (0.51, 0.64) | 0.59 (0.51, 0.67) | 1.4 (1.4, 1.5) |
| Low SDI | Male | Brain and central nervous system cancer | 0.99 (0.93, 1.1) | 1.1 (1, 1.1) | 1.4 (1.3, 1.4) |
| Low SDI | Male | Breast cancer | 0.41 (0.35, 0.46) | 0.42 (0.36, 0.48) | 1.3 (1.2, 1.4) |
| Low SDI | Male | Colon and rectum cancer | 0.5 (0.4, 0.59) | 0.54 (0.44, 0.63) | 1.1 (0.97, 1.2) |
| Low SDI | Male | Esophageal cancer | 0.14 (0.085, 0.2) | 0.18 (0.12, 0.24) | 0.21 (0.15, 0.28) |
| Low SDI | Male | Eye cancer | 0.041 (-0.019, 0.1) | 0.042 (-0.018, 0.1) | 0.84 (0.72, 0.97) |
| Low SDI | Male | Gallbladder and biliary tract cancer | 0.2 (0.017, 0.39) | 0.26 (0.075, 0.44) | 0.44 (0.26, 0.63) |
| Low SDI | Male | Hodgkin lymphoma | -1.3 (-1.4, -1.2) | -1.3 (-1.4, -1.2) | -0.4 (-0.45, -0.35) |
| Low SDI | Male | Kidney cancer | 1.4 (1.3, 1.4) | 1.4 (1.4, 1.5) | 2.3 (2.2, 2.3) |
| Low SDI | Male | Larynx cancer | -0.6 (-0.76, -0.44) | -0.57 (-0.73, -0.41) | -0.059 (-0.24, 0.12) |
| Low SDI | Male | Leukemia | -0.46 (-0.58, -0.35) | -0.38 (-0.48, -0.27) | -0.21 (-0.32, -0.092) |
| Low SDI | Male | Lip and oral cavity cancer | 0.0033 (-0.083, 0.09) | 0.024 (-0.063, 0.11) | 0.88 (0.77, 0.99) |
| Low SDI | Male | Liver cancer | -1.5 (-1.7, -1.2) | -1.4 (-1.7, -1.2) | -1.4 (-1.6, -1.2) |
| Low SDI | Male | Malignant neoplasm of bone and articular cartilage | 0.25 (0.14, 0.37) | 0.28 (0.17, 0.39) | 0.71 (0.58, 0.84) |
| Low SDI | Male | Malignant skin melanoma | 0.38 (0.35, 0.41) | 0.39 (0.37, 0.42) | 1.3 (1.2, 1.4) |
| Low SDI | Male | Mesothelioma | 0.78 (0.7, 0.85) | 0.8 (0.72, 0.88) | 0.87 (0.81, 0.94) |
| Low SDI | Male | Multiple myeloma | 1.7 (1.6, 1.7) | 1.7 (1.6, 1.8) | 2 (1.9, 2.1) |
| Low SDI | Male | Nasopharynx cancer | -0.081 (-0.19, 0.025) | -0.034 (-0.14, 0.073) | 0.28 (0.18, 0.38) |
| Low SDI | Male | Neuroblastoma and other peripheral nervous cell tumors | 4.1 (4, 4.3) | 4.2 (4.1, 4.3) | 4.8 (4.6, 5) |
| Low SDI | Male | Non-Hodgkin lymphoma | -0.22 (-0.3, -0.14) | -0.16 (-0.23, -0.087) | 0.62 (0.49, 0.75) |
| Low SDI | Male | Non-melanoma skin cancer | 1 (0.94, 1.1) | 1 (0.95, 1.1) | 0.24 (0.2, 0.27) |
| Low SDI | Male | Other malignant neoplasms | -0.067 (-0.18, 0.05) | -0.0011 (-0.11, 0.11) | 0.39 (0.25, 0.54) |
| Low SDI | Male | Other pharynx cancer | 0.24 (-0.028, 0.51) | 0.27 (0.01, 0.54) | 0.94 (0.65, 1.2) |
| Low SDI | Male | Pancreatic cancer | 1.4 (1.3, 1.4) | 1.4 (1.4, 1.5) | 1.4 (1.4, 1.5) |
| Low SDI | Male | Prostate cancer | 1.1 (1, 1.2) | 1.1 (0.98, 1.2) | 2.3 (2.3, 2.3) |
| Low SDI | Male | Soft tissue and other extraosseous sarcomas | -0.69 (-0.76, -0.61) | -0.63 (-0.69, -0.56) | 0.0068 (-0.068, 0.082) |
| Low SDI | Male | Stomach cancer | -1.3 (-1.4, -1.2) | -1.2 (-1.4, -1.1) | -1.1 (-1.2, -0.95) |
| Low SDI | Male | Testicular cancer | 1 (0.92, 1.1) | 0.99 (0.89, 1.1) | 3.1 (3, 3.2) |
| Low SDI | Male | Thyroid cancer | 0.71 (0.62, 0.8) | 0.71 (0.62, 0.8) | 2.8 (2.7, 3) |
| Low SDI | Male | Tracheal, bronchus, and lung cancer | 0.13 (0.039, 0.22) | 0.2 (0.1, 0.29) | 0.23 (0.13, 0.32) |
| Low-middle SDI | Both | Bladder cancer | 0.44 (0.33, 0.55) | 0.41 (0.3, 0.52) | 1.4 (1.3, 1.6) |
| Low-middle SDI | Both | Brain and central nervous system cancer | 0.81 (0.71, 0.9) | 0.86 (0.77, 0.94) | 1.3 (1.2, 1.4) |
| Low-middle SDI | Both | Breast cancer | 1.9 (1.7, 2.1) | 1.9 (1.7, 2.1) | 3.1 (3, 3.3) |
| Low-middle SDI | Both | Cervical cancer | -0.51 (-0.82, -0.2) | -0.47 (-0.79, -0.14) | 0.56 (0.25, 0.88) |
| Low-middle SDI | Both | Colon and rectum cancer | 0.54 (0.33, 0.76) | 0.58 (0.36, 0.81) | 1.4 (1.2, 1.6) |
| Low-middle SDI | Both | Esophageal cancer | -0.34 (-0.54, -0.14) | -0.31 (-0.5, -0.11) | -0.19 (-0.39, -0.00015) |
| Low-middle SDI | Both | Eye cancer | 1.2 (1, 1.3) | 1.2 (1.1, 1.3) | 2 (1.8, 2.2) |
| Low-middle SDI | Both | Gallbladder and biliary tract cancer | 0.4 (0.16, 0.65) | 0.44 (0.19, 0.68) | 0.67 (0.43, 0.91) |
| Low-middle SDI | Both | Hodgkin lymphoma | -1.3 (-1.4, -1.2) | -1.2 (-1.3, -1.1) | 0.19 (0.03, 0.35) |
| Low-middle SDI | Both | Kidney cancer | 1.3 (1.1, 1.5) | 1.3 (1.1, 1.5) | 2.3 (2.1, 2.5) |
| Low-middle SDI | Both | Larynx cancer | -0.21 (-0.47, 0.046) | -0.24 (-0.5, 0.015) | 0.43 (0.15, 0.71) |
| Low-middle SDI | Both | Leukemia | -0.71 (-0.79, -0.63) | -0.58 (-0.67, -0.5) | -0.32 (-0.41, -0.23) |
| Low-middle SDI | Both | Lip and oral cavity cancer | 0.93 (0.78, 1.1) | 0.94 (0.79, 1.1) | 2 (1.8, 2.1) |
| Low-middle SDI | Both | Liver cancer | -0.1 (-0.27, 0.064) | -0.071 (-0.23, 0.089) | -0.027 (-0.22, 0.17) |
| Low-middle SDI | Both | Malignant neoplasm of bone and articular cartilage | 0.086 (0.0041, 0.17) | 0.2 (0.12, 0.27) | 0.82 (0.71, 0.93) |
| Low-middle SDI | Both | Malignant skin melanoma | 0.99 (0.85, 1.1) | 1 (0.86, 1.1) | 2.5 (2.4, 2.6) |
| Low-middle SDI | Both | Mesothelioma | 1.2 (1.1, 1.3) | 1.2 (1.1, 1.3) | 1.3 (1.2, 1.4) |
| Low-middle SDI | Both | Multiple myeloma | 2.4 (2.3, 2.6) | 2.4 (2.3, 2.6) | 2.8 (2.7, 3) |
| Low-middle SDI | Both | Nasopharynx cancer | -0.14 (-0.32, 0.047) | -0.061 (-0.25, 0.13) | 0.47 (0.29, 0.65) |
| Low-middle SDI | Both | Neuroblastoma and other peripheral nervous cell tumors | 2.6 (2.5, 2.8) | 2.7 (2.6, 2.9) | 3.4 (3.1, 3.8) |
| Low-middle SDI | Both | Non-Hodgkin lymphoma | 0.2 (0.07, 0.33) | 0.28 (0.15, 0.41) | 1.4 (1.3, 1.6) |
| Low-middle SDI | Both | Non-melanoma skin cancer | 0.64 (0.4, 0.87) | 0.68 (0.45, 0.91) | 1.1 (0.46, 1.8) |
| Low-middle SDI | Both | Other malignant neoplasms | -0.022 (-0.17, 0.13) | 0.079 (-0.074, 0.23) | 0.82 (0.67, 0.98) |
| Low-middle SDI | Both | Other pharynx cancer | 0.86 (0.59, 1.1) | 0.85 (0.58, 1.1) | 1.6 (1.4, 1.9) |
| Low-middle SDI | Both | Ovarian cancer | 2 (1.8, 2.1) | 2 (1.8, 2.1) | 2.2 (2.1, 2.3) |
| Low-middle SDI | Both | Pancreatic cancer | 1.4 (1.3, 1.6) | 1.5 (1.3, 1.6) | 1.5 (1.4, 1.7) |
| Low-middle SDI | Both | Prostate cancer | 1.1 (0.99, 1.1) | 1.1 (1, 1.1) | 1.8 (1.7, 2) |
| Low-middle SDI | Both | Soft tissue and other extraosseous sarcomas | -0.6 (-0.77, -0.43) | -0.47 (-0.64, -0.29) | 0.16 (-0.019, 0.33) |
| Low-middle SDI | Both | Stomach cancer | -1.4 (-1.6, -1.2) | -1.3 (-1.5, -1.2) | -1.1 (-1.3, -0.92) |
| Low-middle SDI | Both | Testicular cancer | 1.2 (0.95, 1.5) | 1.2 (0.96, 1.5) | 3.7 (3.4, 3.9) |
| Low-middle SDI | Both | Thyroid cancer | 0.41 (0.21, 0.61) | 0.37 (0.17, 0.57) | 2.5 (2.3, 2.7) |
| Low-middle SDI | Both | Tracheal, bronchus, and lung cancer | 0.22 (0.082, 0.36) | 0.23 (0.09, 0.37) | 0.31 (0.17, 0.45) |
| Low-middle SDI | Both | Uterine cancer | 0.83 (0.66, 1) | 0.81 (0.63, 0.99) | 2.2 (2.1, 2.4) |
| Low-middle SDI | Female | Bladder cancer | 0.48 (0.39, 0.56) | 0.5 (0.42, 0.59) | 1.4 (1.3, 1.5) |
| Low-middle SDI | Female | Brain and central nervous system cancer | 0.91 (0.79, 1) | 0.93 (0.83, 1) | 1.5 (1.4, 1.6) |
| Low-middle SDI | Female | Breast cancer | 1.9 (1.7, 2.1) | 1.9 (1.7, 2.1) | 3.1 (3, 3.3) |
| Low-middle SDI | Female | Cervical cancer | -0.51 (-0.82, -0.2) | -0.47 (-0.8, -0.14) | 0.56 (0.25, 0.87) |
| Low-middle SDI | Female | Colon and rectum cancer | 0.49 (0.21, 0.77) | 0.55 (0.24, 0.87) | 1.4 (1.2, 1.7) |
| Low-middle SDI | Female | Esophageal cancer | -0.81 (-1.1, -0.55) | -0.75 (-1, -0.49) | -0.59 (-0.85, -0.33) |
| Low-middle SDI | Female | Eye cancer | 1.5 (1.4, 1.7) | 1.6 (1.4, 1.8) | 2.4 (2.2, 2.7) |
| Low-middle SDI | Female | Gallbladder and biliary tract cancer | 0.37 (0.058, 0.69) | 0.43 (0.1, 0.75) | 0.62 (0.3, 0.94) |
| Low-middle SDI | Female | Hodgkin lymphoma | -1.4 (-1.6, -1.2) | -1.3 (-1.5, -1.1) | 0.25 (-0.041, 0.55) |
| Low-middle SDI | Female | Kidney cancer | 1 (0.86, 1.2) | 1.1 (0.94, 1.3) | 2.4 (2.2, 2.6) |
| Low-middle SDI | Female | Larynx cancer | 0.4 (0.18, 0.62) | 0.43 (0.2, 0.67) | 1.1 (0.87, 1.3) |
| Low-middle SDI | Female | Leukemia | -0.89 (-1, -0.77) | -0.75 (-0.87, -0.64) | -0.51 (-0.63, -0.39) |
| Low-middle SDI | Female | Lip and oral cavity cancer | 1.2 (0.98, 1.5) | 1.3 (1, 1.6) | 2.4 (2.2, 2.7) |
| Low-middle SDI | Female | Liver cancer | 0.01 (-0.12, 0.14) | 0.083 (-0.031, 0.2) | 0.066 (-0.079, 0.21) |
| Low-middle SDI | Female | Malignant neoplasm of bone and articular cartilage | -0.45 (-0.62, -0.27) | -0.3 (-0.45, -0.15) | 0.33 (0.17, 0.48) |
| Low-middle SDI | Female | Malignant skin melanoma | 1.3 (1.1, 1.4) | 1.3 (1.1, 1.4) | 3 (2.9, 3.1) |
| Low-middle SDI | Female | Mesothelioma | 1.7 (1.6, 1.8) | 1.7 (1.6, 1.8) | 1.9 (1.8, 2) |
| Low-middle SDI | Female | Multiple myeloma | 2.6 (2.5, 2.8) | 2.6 (2.5, 2.8) | 3 (2.9, 3.2) |
| Low-middle SDI | Female | Nasopharynx cancer | -0.49 (-0.76, -0.21) | -0.36 (-0.65, -0.068) | 0.26 (-0.0085, 0.53) |
| Low-middle SDI | Female | Neuroblastoma and other peripheral nervous cell tumors | 2.5 (2.3, 2.7) | 2.6 (2.4, 2.8) | 3.4 (3.1, 3.7) |
| Low-middle SDI | Female | Non-Hodgkin lymphoma | 0.69 (0.57, 0.81) | 0.8 (0.68, 0.91) | 2 (1.8, 2.1) |
| Low-middle SDI | Female | Non-melanoma skin cancer | 0.3 (0.056, 0.55) | 0.37 (0.14, 0.61) | 1.3 (0.49, 2) |
| Low-middle SDI | Female | Other malignant neoplasms | -0.011 (-0.21, 0.18) | 0.11 (-0.096, 0.33) | 0.96 (0.78, 1.1) |
| Low-middle SDI | Female | Other pharynx cancer | 0.83 (0.63, 1) | 0.83 (0.63, 1) | 1.8 (1.6, 2) |
| Low-middle SDI | Female | Ovarian cancer | 2 (1.8, 2.1) | 2 (1.8, 2.1) | 2.2 (2.1, 2.3) |
| Low-middle SDI | Female | Pancreatic cancer | 1.3 (1.2, 1.5) | 1.4 (1.2, 1.5) | 1.5 (1.3, 1.6) |
| Low-middle SDI | Female | Soft tissue and other extraosseous sarcomas | -0.7 (-0.87, -0.54) | -0.55 (-0.71, -0.4) | 0.071 (-0.09, 0.23) |
| Low-middle SDI | Female | Stomach cancer | -1.3 (-1.6, -1) | -1.2 (-1.5, -0.91) | -1 (-1.3, -0.71) |
| Low-middle SDI | Female | Thyroid cancer | 0.12 (-0.13, 0.37) | 0.054 (-0.2, 0.31) | 2.3 (2.1, 2.6) |
| Low-middle SDI | Female | Tracheal, bronchus, and lung cancer | 1.3 (1, 1.5) | 1.3 (1.1, 1.6) | 1.4 (1.1, 1.7) |
| Low-middle SDI | Female | Uterine cancer | 0.83 (0.66, 1) | 0.81 (0.63, 0.99) | 2.2 (2.1, 2.4) |
| Low-middle SDI | Male | Bladder cancer | 0.42 (0.27, 0.57) | 0.37 (0.22, 0.52) | 1.5 (1.2, 1.7) |
| Low-middle SDI | Male | Brain and central nervous system cancer | 0.73 (0.63, 0.82) | 0.8 (0.71, 0.9) | 1.2 (1.1, 1.3) |
| Low-middle SDI | Male | Breast cancer | 0.92 (0.79, 1.1) | 0.92 (0.78, 1.1) | 2.1 (1.9, 2.2) |
| Low-middle SDI | Male | Colon and rectum cancer | 0.6 (0.43, 0.77) | 0.62 (0.44, 0.79) | 1.4 (1.2, 1.5) |
| Low-middle SDI | Male | Esophageal cancer | -0.0079 (-0.26, 0.24) | -0.00052 (-0.24, 0.24) | 0.087 (-0.16, 0.33) |
| Low-middle SDI | Male | Eye cancer | 0.71 (0.6, 0.81) | 0.76 (0.66, 0.86) | 1.5 (1.3, 1.6) |
| Low-middle SDI | Male | Gallbladder and biliary tract cancer | 0.45 (0.28, 0.63) | 0.46 (0.28, 0.63) | 0.75 (0.57, 0.92) |
| Low-middle SDI | Male | Hodgkin lymphoma | -1.2 (-1.3, -1.1) | -1.1 (-1.2, -1) | 0.15 (0.051, 0.24) |
| Low-middle SDI | Male | Kidney cancer | 1.4 (1.2, 1.6) | 1.4 (1.2, 1.6) | 2.3 (2.1, 2.5) |
| Low-middle SDI | Male | Larynx cancer | -0.37 (-0.68, -0.063) | -0.4 (-0.7, -0.099) | 0.27 (-0.049, 0.6) |
| Low-middle SDI | Male | Leukemia | -0.54 (-0.62, -0.45) | -0.43 (-0.52, -0.33) | -0.15 (-0.25, -0.058) |
| Low-middle SDI | Male | Lip and oral cavity cancer | 0.74 (0.56, 0.92) | 0.73 (0.56, 0.91) | 1.7 (1.5, 1.8) |
| Low-middle SDI | Male | Liver cancer | -0.16 (-0.36, 0.044) | -0.14 (-0.34, 0.055) | -0.072 (-0.29, 0.15) |
| Low-middle SDI | Male | Malignant neoplasm of bone and articular cartilage | 0.44 (0.31, 0.56) | 0.52 (0.4, 0.64) | 1.1 (0.99, 1.3) |
| Low-middle SDI | Male | Malignant skin melanoma | 0.69 (0.54, 0.85) | 0.73 (0.58, 0.88) | 1.8 (1.7, 2) |
| Low-middle SDI | Male | Mesothelioma | 0.88 (0.78, 0.99) | 0.89 (0.79, 1) | 0.99 (0.88, 1.1) |
| Low-middle SDI | Male | Multiple myeloma | 2.3 (2.1, 2.5) | 2.3 (2.1, 2.5) | 2.7 (2.5, 2.9) |
| Low-middle SDI | Male | Nasopharynx cancer | 0.064 (-0.098, 0.23) | 0.1 (-0.061, 0.27) | 0.58 (0.42, 0.74) |
| Low-middle SDI | Male | Neuroblastoma and other peripheral nervous cell tumors | 2.7 (2.5, 2.9) | 2.9 (2.7, 3) | 3.5 (3.2, 3.8) |
| Low-middle SDI | Male | Non-Hodgkin lymphoma | -0.14 (-0.3, 0.013) | -0.067 (-0.23, 0.1) | 1.1 (0.86, 1.2) |
| Low-middle SDI | Male | Non-melanoma skin cancer | 0.91 (0.66, 1.1) | 0.93 (0.68, 1.2) | 0.94 (0.42, 1.5) |
| Low-middle SDI | Male | Other malignant neoplasms | -0.032 (-0.19, 0.13) | 0.047 (-0.12, 0.21) | 0.68 (0.5, 0.87) |
| Low-middle SDI | Male | Other pharynx cancer | 0.87 (0.55, 1.2) | 0.86 (0.54, 1.2) | 1.6 (1.3, 1.9) |
| Low-middle SDI | Male | Pancreatic cancer | 1.5 (1.4, 1.6) | 1.5 (1.4, 1.7) | 1.6 (1.4, 1.7) |
| Low-middle SDI | Male | Prostate cancer | 1.1 (0.99, 1.1) | 1.1 (1, 1.1) | 1.8 (1.7, 2) |
| Low-middle SDI | Male | Soft tissue and other extraosseous sarcomas | -0.5 (-0.71, -0.29) | -0.38 (-0.6, -0.16) | 0.24 (0.026, 0.45) |
| Low-middle SDI | Male | Stomach cancer | -1.5 (-1.6, -1.3) | -1.5 (-1.6, -1.3) | -1.2 (-1.4, -1) |
| Low-middle SDI | Male | Testicular cancer | 1.2 (0.96, 1.5) | 1.2 (0.96, 1.5) | 3.7 (3.4, 3.9) |
| Low-middle SDI | Male | Thyroid cancer | 1.1 (0.92, 1.2) | 1 (0.89, 1.2) | 3.2 (3.1, 3.3) |
| Low-middle SDI | Male | Tracheal, bronchus, and lung cancer | -0.34 (-0.51, -0.16) | -0.33 (-0.51, -0.16) | -0.26 (-0.43, -0.079) |
| Middle SDI | Both | Bladder cancer | -0.46 (-0.62, -0.29) | -0.6 (-0.75, -0.44) | 1 (0.85, 1.2) |
| Middle SDI | Both | Brain and central nervous system cancer | -0.035 (-0.07, -0.00063) | -0.0036 (-0.041, 0.034) | 0.92 (0.84, 0.99) |
| Middle SDI | Both | Breast cancer | 1.6 (1.5, 1.6) | 1.5 (1.4, 1.5) | 3 (2.9, 3.1) |
| Middle SDI | Both | Cervical cancer | -0.0068 (-0.073, 0.059) | -0.046 (-0.12, 0.025) | 1.2 (1.1, 1.3) |
| Middle SDI | Both | Colon and rectum cancer | 0.39 (0.31, 0.46) | 0.31 (0.23, 0.38) | 1.8 (1.7, 2) |
| Middle SDI | Both | Esophageal cancer | -2.1 (-2.6, -1.6) | -2.2 (-2.7, -1.7) | -1.8 (-2.4, -1.3) |
| Middle SDI | Both | Eye cancer | -0.22 (-0.29, -0.14) | -0.24 (-0.31, -0.17) | 0.61 (0.5, 0.73) |
| Middle SDI | Both | Gallbladder and biliary tract cancer | -0.07 (-0.15, 0.0051) | -0.064 (-0.14, 0.012) | 0.74 (0.63, 0.85) |
| Middle SDI | Both | Hodgkin lymphoma | -1.5 (-1.6, -1.4) | -1.4 (-1.5, -1.3) | 1 (0.87, 1.1) |
| Middle SDI | Both | Kidney cancer | 0.86 (0.75, 0.96) | 0.82 (0.71, 0.93) | 2.1 (1.9, 2.2) |
| Middle SDI | Both | Larynx cancer | -0.86 (-1.1, -0.57) | -0.89 (-1.2, -0.6) | 0.32 (0.048, 0.59) |
| Middle SDI | Both | Leukemia | -1 (-1.1, -0.92) | -0.94 (-1, -0.84) | -0.29 (-0.4, -0.18) |
| Middle SDI | Both | Lip and oral cavity cancer | 0.71 (0.64, 0.78) | 0.7 (0.63, 0.78) | 2 (2, 2.1) |
| Middle SDI | Both | Liver cancer | -0.93 (-1.4, -0.42) | -1 (-1.6, -0.46) | -0.58 (-1.1, -0.056) |
| Middle SDI | Both | Malignant neoplasm of bone and articular cartilage | -0.38 (-0.5, -0.26) | -0.31 (-0.44, -0.19) | 0.54 (0.41, 0.67) |
| Middle SDI | Both | Malignant skin melanoma | 0.091 (-0.0018, 0.18) | 0.011 (-0.088, 0.11) | 2.4 (2.3, 2.6) |
| Middle SDI | Both | Mesothelioma | -0.23 (-0.46, -0.0095) | -0.25 (-0.48, -0.024) | -0.066 (-0.3, 0.17) |
| Middle SDI | Both | Multiple myeloma | 2.2 (2.1, 2.3) | 2.2 (2.1, 2.4) | 2.9 (2.8, 3) |
| Middle SDI | Both | Nasopharynx cancer | -0.89 (-0.95, -0.83) | -0.97 (-1, -0.9) | 1.3 (1.1, 1.5) |
| Middle SDI | Both | Neuroblastoma and other peripheral nervous cell tumors | 2.1 (2, 2.2) | 2.2 (2.1, 2.3) | 2.7 (2.6, 2.8) |
| Middle SDI | Both | Non-Hodgkin lymphoma | -0.094 (-0.19, 0.00023) | -0.12 (-0.21, -0.035) | 1.9 (1.8, 2.1) |
| Middle SDI | Both | Non-melanoma skin cancer | -0.97 (-1.1, -0.85) | -1 (-1.1, -0.92) | 3.4 (1.8, 5) |
| Middle SDI | Both | Other malignant neoplasms | 0.15 (0.079, 0.22) | 0.21 (0.13, 0.28) | 1.9 (1.7, 2) |
| Middle SDI | Both | Other pharynx cancer | 1.3 (1.2, 1.5) | 1.3 (1.2, 1.5) | 2.6 (2.4, 2.7) |
| Middle SDI | Both | Ovarian cancer | 1.6 (1.6, 1.7) | 1.7 (1.6, 1.7) | 1.9 (1.8, 1.9) |
| Middle SDI | Both | Pancreatic cancer | 0.64 (0.56, 0.71) | 0.59 (0.52, 0.66) | 0.73 (0.65, 0.8) |
| Middle SDI | Both | Prostate cancer | 0.65 (0.56, 0.74) | 0.52 (0.43, 0.62) | 2.5 (2.4, 2.7) |
| Middle SDI | Both | Soft tissue and other extraosseous sarcomas | 0.073 (-0.047, 0.19) | 0.2 (0.084, 0.31) | 0.84 (0.71, 0.97) |
| Middle SDI | Both | Stomach cancer | -2.6 (-2.8, -2.4) | -2.8 (-3, -2.5) | -2.1 (-2.3, -1.8) |
| Middle SDI | Both | Testicular cancer | 2.2 (1.8, 2.7) | 2.2 (1.8, 2.6) | 4.9 (4.3, 5.5) |
| Middle SDI | Both | Thyroid cancer | 0.66 (0.56, 0.76) | 0.44 (0.35, 0.54) | 2.7 (2.5, 2.8) |
| Middle SDI | Both | Tracheal, bronchus, and lung cancer | -1.1 (-1.2, -0.95) | -1.2 (-1.3, -1) | -0.83 (-0.98, -0.67) |
| Middle SDI | Both | Uterine cancer | -0.99 (-1.5, -0.44) | -1.1 (-1.6, -0.55) | 0.6 (-0.018, 1.2) |
| Middle SDI | Female | Bladder cancer | -0.21 (-0.46, 0.038) | -0.25 (-0.48, -0.016) | 1 (0.73, 1.3) |
| Middle SDI | Female | Brain and central nervous system cancer | -0.13 (-0.21, -0.036) | -0.086 (-0.17, 0.0028) | 0.9 (0.78, 1) |
| Middle SDI | Female | Breast cancer | 1.7 (1.6, 1.7) | 1.6 (1.5, 1.7) | 3.2 (3, 3.3) |
| Middle SDI | Female | Cervical cancer | 0.076 (0.0095, 0.14) | 0.037 (-0.032, 0.11) | 1.3 (1.2, 1.4) |
| Middle SDI | Female | Colon and rectum cancer | 0.38 (0.25, 0.5) | 0.39 (0.27, 0.51) | 1.7 (1.5, 1.9) |
| Middle SDI | Female | Esophageal cancer | -1.3 (-1.8, -0.9) | -1.3 (-1.7, -0.87) | -1.2 (-1.7, -0.63) |
| Middle SDI | Female | Eye cancer | -0.24 (-0.36, -0.13) | -0.23 (-0.34, -0.12) | 0.52 (0.34, 0.69) |
| Middle SDI | Female | Gallbladder and biliary tract cancer | 0.28 (0.12, 0.43) | 0.33 (0.18, 0.48) | 0.87 (0.7, 1) |
| Middle SDI | Female | Hodgkin lymphoma | -1.4 (-1.5, -1.2) | -1.2 (-1.4, -1.1) | 1.2 (1.1, 1.4) |
| Middle SDI | Female | Kidney cancer | 1.3 (1.1, 1.5) | 1.4 (1.2, 1.6) | 2.6 (2.4, 2.9) |
| Middle SDI | Female | Larynx cancer | -0.33 (-0.63, -0.034) | -0.24 (-0.55, 0.061) | 0.87 (0.55, 1.2) |
| Middle SDI | Female | Leukemia | -1.4 (-1.5, -1.2) | -1.2 (-1.4, -1.1) | -0.65 (-0.81, -0.49) |
| Middle SDI | Female | Lip and oral cavity cancer | 1.7 (1.6, 1.8) | 1.8 (1.7, 1.9) | 3 (2.9, 3.2) |
| Middle SDI | Female | Liver cancer | -0.69 (-0.97, -0.41) | -0.73 (-1.1, -0.41) | -0.3 (-0.51, -0.083) |
| Middle SDI | Female | Malignant neoplasm of bone and articular cartilage | -0.81 (-0.91, -0.71) | -0.65 (-0.74, -0.56) | 0.16 (0.12, 0.2) |
| Middle SDI | Female | Malignant skin melanoma | 0.34 (0.21, 0.48) | 0.28 (0.14, 0.43) | 2.7 (2.5, 2.8) |
| Middle SDI | Female | Mesothelioma | 0.22 (-0.007, 0.46) | 0.25 (0.0055, 0.49) | 0.52 (0.28, 0.77) |
| Middle SDI | Female | Multiple myeloma | 2.1 (2, 2.2) | 2.1 (2, 2.3) | 2.8 (2.6, 2.9) |
| Middle SDI | Female | Nasopharynx cancer | -1.2 (-1.4, -0.94) | -1.1 (-1.3, -0.93) | 0.82 (0.51, 1.1) |
| Middle SDI | Female | Neuroblastoma and other peripheral nervous cell tumors | 1.9 (1.8, 2) | 2 (2, 2.1) | 2.5 (2.4, 2.6) |
| Middle SDI | Female | Non-Hodgkin lymphoma | 0.39 (0.19, 0.58) | 0.46 (0.28, 0.64) | 2.5 (2.2, 2.7) |
| Middle SDI | Female | Non-melanoma skin cancer | -1.5 (-1.7, -1.3) | -1.6 (-1.8, -1.4) | 4 (2.2, 5.8) |
| Middle SDI | Female | Other malignant neoplasms | 0.47 (0.37, 0.56) | 0.6 (0.49, 0.71) | 2.2 (2.1, 2.4) |
| Middle SDI | Female | Other pharynx cancer | 1.4 (1.2, 1.5) | 1.5 (1.4, 1.6) | 2.9 (2.8, 3) |
| Middle SDI | Female | Ovarian cancer | 1.7 (1.7, 1.8) | 1.8 (1.7, 1.8) | 2 (1.9, 2) |
| Middle SDI | Female | Pancreatic cancer | 0.78 (0.66, 0.9) | 0.8 (0.69, 0.92) | 0.93 (0.81, 1) |
| Middle SDI | Female | Soft tissue and other extraosseous sarcomas | 0.16 (-0.019, 0.33) | 0.34 (0.16, 0.51) | 0.93 (0.75, 1.1) |
| Middle SDI | Female | Stomach cancer | -2.1 (-2.4, -1.8) | -2.1 (-2.3, -1.8) | -1.6 (-1.9, -1.4) |
| Middle SDI | Female | Thyroid cancer | 0.75 (0.62, 0.88) | 0.53 (0.41, 0.65) | 2.7 (2.5, 2.8) |
| Middle SDI | Female | Tracheal, bronchus, and lung cancer | -0.087 (-0.31, 0.14) | -0.087 (-0.3, 0.13) | 0.35 (0.11, 0.6) |
| Middle SDI | Female | Uterine cancer | -0.91 (-1.5, -0.35) | -1 (-1.6, -0.46) | 0.69 (0.056, 1.3) |
| Middle SDI | Male | Bladder cancer | -0.59 (-0.73, -0.46) | -0.77 (-0.9, -0.63) | 0.99 (0.82, 1.2) |
| Middle SDI | Male | Brain and central nervous system cancer | 0.0079 (-0.033, 0.048) | 0.033 (-0.00047, 0.066) | 0.92 (0.85, 0.99) |
| Middle SDI | Male | Breast cancer | -1.1 (-1.2, -0.99) | -1.3 (-1.4, -1.2) | -0.022 (-0.29, 0.24) |
| Middle SDI | Male | Colon and rectum cancer | 0.36 (0.27, 0.44) | 0.22 (0.14, 0.31) | 1.8 (1.7, 2) |
| Middle SDI | Male | Esophageal cancer | -2.4 (-2.9, -1.8) | -2.5 (-3, -2) | -2 (-2.6, -1.5) |
| Middle SDI | Male | Eye cancer | -0.2 (-0.33, -0.064) | -0.26 (-0.38, -0.14) | 0.7 (0.52, 0.88) |
| Middle SDI | Male | Gallbladder and biliary tract cancer | -0.4 (-0.54, -0.27) | -0.44 (-0.59, -0.29) | 0.62 (0.45, 0.79) |
| Middle SDI | Male | Hodgkin lymphoma | -1.6 (-1.8, -1.5) | -1.6 (-1.7, -1.4) | 0.81 (0.66, 0.97) |
| Middle SDI | Male | Kidney cancer | 0.63 (0.5, 0.76) | 0.56 (0.43, 0.69) | 1.8 (1.6, 1.9) |
| Middle SDI | Male | Larynx cancer | -1 (-1.3, -0.75) | -1.1 (-1.4, -0.8) | 0.14 (-0.12, 0.41) |
| Middle SDI | Male | Leukemia | -0.79 (-0.91, -0.67) | -0.75 (-0.86, -0.64) | -0.051 (-0.17, 0.071) |
| Middle SDI | Male | Lip and oral cavity cancer | 0.34 (0.26, 0.41) | 0.29 (0.21, 0.37) | 1.6 (1.5, 1.6) |
| Middle SDI | Male | Liver cancer | -1 (-1.6, -0.47) | -1.1 (-1.8, -0.53) | -0.7 (-1.3, -0.11) |
| Middle SDI | Male | Malignant neoplasm of bone and articular cartilage | -0.16 (-0.36, 0.038) | -0.14 (-0.34, 0.052) | 0.74 (0.54, 0.94) |
| Middle SDI | Male | Malignant skin melanoma | -0.12 (-0.26, 0.019) | -0.21 (-0.34, -0.081) | 2.1 (1.9, 2.3) |
| Middle SDI | Male | Mesothelioma | -0.51 (-0.74, -0.28) | -0.54 (-0.78, -0.31) | -0.4 (-0.65, -0.16) |
| Middle SDI | Male | Multiple myeloma | 2.3 (2.1, 2.5) | 2.3 (2.1, 2.4) | 3 (2.8, 3.1) |
| Middle SDI | Male | Nasopharynx cancer | -0.84 (-0.95, -0.73) | -0.96 (-1.1, -0.84) | 1.5 (1.3, 1.6) |
| Middle SDI | Male | Neuroblastoma and other peripheral nervous cell tumors | 2.2 (2, 2.4) | 2.3 (2.1, 2.5) | 2.8 (2.6, 2.9) |
| Middle SDI | Male | Non-Hodgkin lymphoma | -0.39 (-0.48, -0.3) | -0.47 (-0.56, -0.38) | 1.6 (1.4, 1.8) |
| Middle SDI | Male | Non-melanoma skin cancer | -0.53 (-0.63, -0.43) | -0.57 (-0.66, -0.48) | 2.7 (1.3, 4.1) |
| Middle SDI | Male | Other malignant neoplasms | -0.11 (-0.2, -0.016) | -0.1 (-0.19, -0.02) | 1.5 (1.4, 1.7) |
| Middle SDI | Male | Other pharynx cancer | 1.2 (1.1, 1.4) | 1.3 (1.1, 1.4) | 2.4 (2.3, 2.6) |
| Middle SDI | Male | Pancreatic cancer | 0.53 (0.46, 0.6) | 0.45 (0.37, 0.52) | 0.59 (0.51, 0.67) |
| Middle SDI | Male | Prostate cancer | 0.57 (0.48, 0.66) | 0.44 (0.35, 0.53) | 2.4 (2.3, 2.6) |
| Middle SDI | Male | Soft tissue and other extraosseous sarcomas | -0.0086 (-0.18, 0.16) | 0.067 (-0.087, 0.22) | 0.76 (0.58, 0.94) |
| Middle SDI | Male | Stomach cancer | -2.9 (-3.1, -2.7) | -3.1 (-3.3, -2.9) | -2.3 (-2.5, -2.1) |
| Middle SDI | Male | Testicular cancer | 2.2 (1.7, 2.6) | 2.1 (1.7, 2.5) | 4.8 (4.2, 5.4) |
| Middle SDI | Male | Thyroid cancer | 0.57 (0.43, 0.7) | 0.36 (0.23, 0.48) | 2.8 (2.5, 3) |
| Middle SDI | Male | Tracheal, bronchus, and lung cancer | -1.6 (-1.8, -1.5) | -1.7 (-1.9, -1.6) | -1.5 (-1.6, -1.3) |


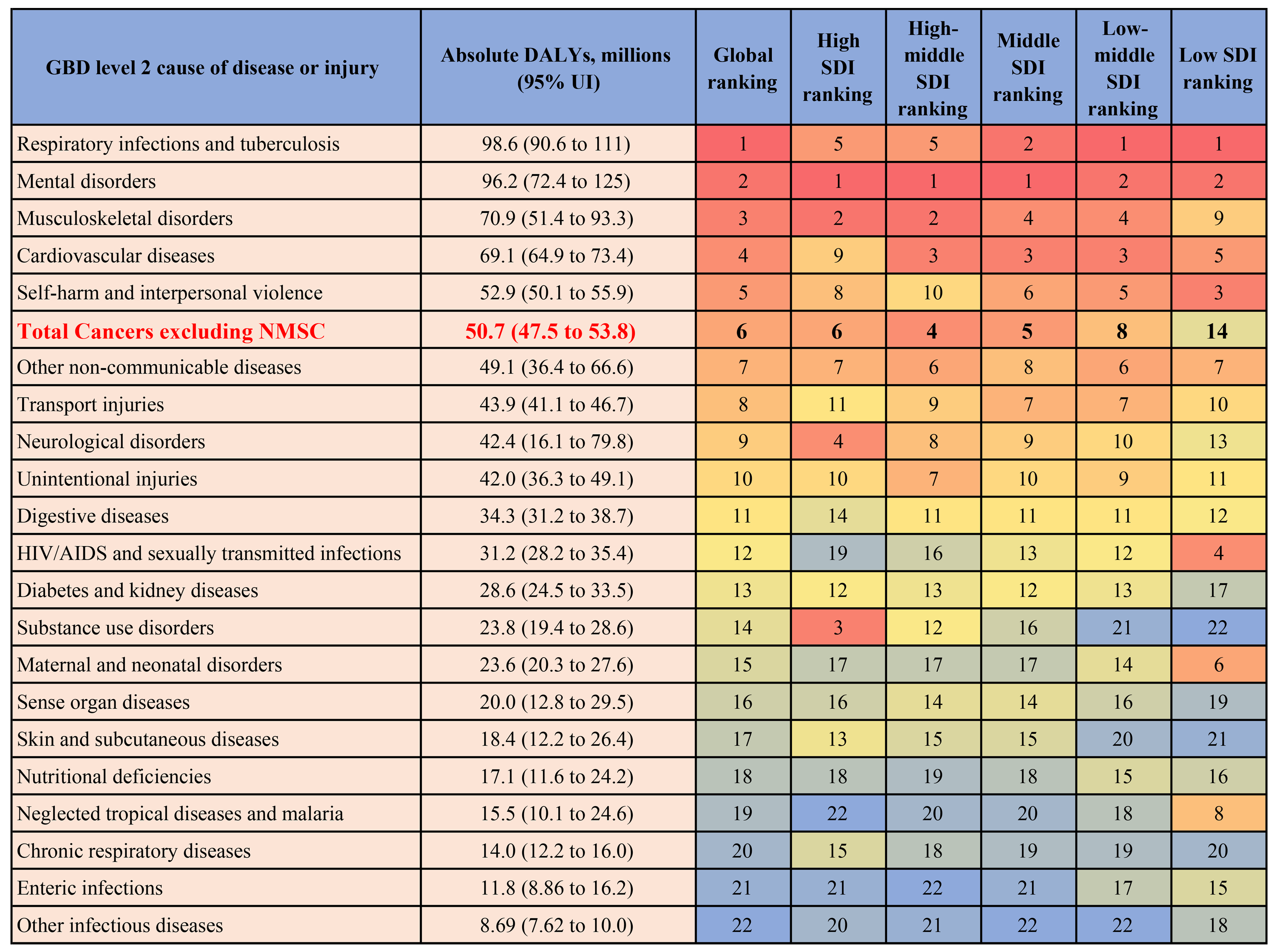


### Figure S1. ﻿Ranking of total cancer absolute disability-adjusted life years (DALYs) in 2021 among the 22 level 2 categories of disease in the Global Burden of Disease (GBD) by quintile of sociodemographic index (SDI)
